# Proteolysis‐Targeting Chimera (PROTAC): Current Applications and Future Directions

**DOI:** 10.1002/mco2.70401

**Published:** 2025-10-04

**Authors:** Gang Fan, Shilin Chen, Qingping Zhang, Na Yu, Ziyang Shen, Zhaoji Liu, Weiming Guo, Zhihan Tang, Jing Yang, Miao Liu

**Affiliations:** ^1^ Medical Research Center Affiliated Nanshan Hospital of Shenzhen University Shenzhen China; ^2^ Pan‐Vascular Research Group Affiliated Nanshan Hospital of Shenzhen University Shenzhen China; ^3^ Department of Endocrinology Affiliated Nanshan Hospital of Shenzhen University Shenzhen China; ^4^ Department of Neurosurgery Affiliated Nanshan Hospital of Shenzhen University Shenzhen China; ^5^ Department of Biology Boston University Boston Massachusetts USA; ^6^ Department of Pathology Brigham and Women's Hospital Harvard Medical School Boston Massachusetts USA; ^7^ Department of Medicine Beth Israel Deaconess Medical Center Boston Massachusetts USA; ^8^ Department of Orthopaedics The First Affiliated Hospital of Guilin Medical University Guilin China; ^9^ Institute of Cardiovascular Disease Key Laboratory for Arteriosclerology of Hunan Province Hunan International Scientific and Technological Cooperation Base of Arteriosclerotic Disease School of Basic Medical Sciences Hengyang Medical School University of South China Hengyang Hunan China; ^10^ School of Basic Medical Sciences Hunan University of Medicine Huaihua China

**Keywords:** clinical translation, drug discovery, PROTAC, target protein degradation, ubiquitin–proteasome system

## Abstract

Targeted protein degradation (TPD) represents a paradigm shift in drug discovery, moving beyond traditional binding‐based inhibition toward active removal of disease‐driving proteins. This approach has unlocked therapeutic possibilities for previously “undruggable” targets, including transcription factors like MYC and STAT3, mutant oncoproteins such as KRAS G12C, and scaffolding molecules lacking conventional binding pockets. Among TPD strategies, proteolysis‐targeting chimeras (PROTACs) have emerged as the leading clinical platform, with the first molecule entering trials in 2019 and progression to Phase III completion by 2024. This comprehensive review examines PROTAC development across diverse therapeutic areas, analyzing key targets including kinases, hormone receptors, antiapoptotic proteins, and epigenetic modulators. We evaluate clinical progression of breakthrough candidates such as ARV‐110 for prostate cancer, ARV‐471 for breast cancer, and BTK degraders, while discussing critical challenges including the “hook effect” and oral bioavailability limitations. The review explores future directions encompassing innovative delivery strategies, tissue‐specific degrader design, and approaches for expanding E3 ligase repertoires and overcoming resistance. This review provides essential foundations for rational target selection, molecular optimization, and clinical translation strategies. By integrating mechanistic insights with clinical realities, this analysis offers perspectives on PROTAC technology advancement and identifies opportunities for transforming treatment of complex diseases resistant to conventional therapies.

## Graphical abstract

PROTACs are reshaping drug discovery through targeted protein degradation. Clinical programs now extend to prostate, breast, lung, hematologic, and solid tumors, with key targets including AR, ER, BTK, STAT3, Bcl‐xL, BRD9, Kras, and so on. These degraders overcome resistance, broaden therapeutic scope, and provide solutions for “undruggable” proteins, advancing from molecular design to Phase III trials and opening opportunities across oncology, immunology, neurology, and metabolic disease.

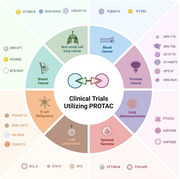

## 1 Introduction

Recent years have witnessed remarkable advances in proteomics and structural biology that have significantly deepened our understanding of disease‐associated molecular mechanisms, yet these developments have simultaneously exposed critical limitations inherent in conventional drug discovery approaches. While small‐molecule inhibitors [1], agonists [2], and monoclonal antibodies (mAbs) [3] have found widespread application in clinical therapeutics, these modalities typically operate through occupancy‐based binding to active sites or allosteric pockets to block protein function or downstream signaling pathways. However, many pathogenic proteins, including transcription factors, structural scaffolding proteins, and intracellular aggregates, lack well‐defined binding pockets, exhibit high conformational plasticity, or display functional redundancy, characteristics that have long rendered them “undruggable”[4, 5, 6, 7]. Current estimates suggest that only 10–15% of the human proteome remains accessible to conventional small‐molecule approaches [8], leaving vast territories of disease‐relevant biology beyond the reach of therapeutic intervention. Against this backdrop, the emergence of targeted protein degradation (TPD) strategies represents a fundamental paradigm shift in drug discovery. Rather than attempting to inhibit protein activity, TPD technologies harness the cell's intrinsic protein quality control machinery to induce proteolytic elimination of the target protein [9, 10, 11, 12]. Among TPD strategies, proteolysis‐targeting chimeras (PROTACs) have emerged as a particularly mature platform. Originally conceived in 2001 as experimental tools for studying protein function [13], PROTACs have undergone rapid evolution into promising clinical candidates. The extraordinary promise of these novel therapeutic approaches has been dramatically demonstrated through recent clinical milestones: the first PROTAC molecule entered clinical trials in 2019 [14], and remarkably, just 5 years later, the field has achieved an even more significant milestone with a PROTAC molecule successfully completing Phase III clinical trials and formally submitting a New Drug Application to the United States Food and Drug Administration (US FDA) for market approval [15].

Clinical validation of PROTAC technology has been most compelling in oncology, where conventional approaches have repeatedly failed. For example, androgen receptor (AR) variants that drive resistance to standard antagonists remain susceptible to degradation‐based strategies [16, 17], and transcription factors such as STAT3—long considered among the most challenging cancer targets—are now tractable through systematic degradation [18]. These successes in cancer have established PROTACs as a leading therapeutic modality. Building on this foundation, research has begun to explore applications beyond oncology, including neurodegenerative diseases [19, 20], metabolic disorders [21], inflammatory conditions [22], and, more recently, cellular senescence [23]. Although senescence‐related targets are still at an early stage of investigation, innovative PROTAC designs have demonstrated that proteins central to senescence, a process increasingly recognized as critical in aging and age‐related diseases, can be effectively degraded [23, 24, 25, 26]. Each therapeutic area presents unique challenges in target selection, molecular design, and delivery, yet most current literature remains fragmented, with cancer‐focused reports dominating and limited synthesis across disease contexts. This review aims to bridge that gap by providing an integrated analytical framework that captures both the well‐established oncology applications and the emerging potential of PROTACs in noncancer indications.

The challenges confronting PROTAC technology are as substantial as its promise. Molecular weight and polarity constraints continue to limit oral bioavailability and tissue distribution profiles. The “hook effect,” whereby higher concentrations paradoxically reduce activity, complicates dose optimization strategies [27, 28]. Resistance mechanisms, while less prevalent than with traditional inhibitors, are beginning to emerge as clinical experience accumulates. Perhaps most critically, the field currently lacks robust predictive frameworks for identifying which proteins will prove amenable to degradation and which patients will respond favorably to specific PROTAC therapies. To address these gaps, we provide a comprehensive analysis that contextualizes PROTAC development across disease systems, examines both successful applications and persistent challenges, and identifies emerging opportunities for therapeutic innovation. Our review encompasses several key dimensions: the mechanistic foundations and pharmacological advantages that distinguish PROTACs from conventional therapies; representative targets and molecules across major therapeutic areas, highlighting both breakthrough successes and instructive failures; current clinical landscape analysis that draws lessons from early trials; innovative delivery approaches that expand PROTAC accessibility to previously challenging targets and tissues; and forward‐looking directions for technology advancement, including strategies for overcoming current limitations and expanding therapeutic applications.

This comprehensive review seeks to provide a systematic analytical framework for understanding PROTAC technology development. We begin by examining the design principles and degradation mechanisms that underpin PROTAC function, followed by a systematic comparison with traditional therapeutic modalities to establish their unique pharmacological advantages. Subsequently, we provide comprehensive coverage of representative targets and molecules across diverse therapeutic areas, ranging from well‐established kinase and hormone receptor degraders to emerging applications targeting transcription factors and immune regulatory proteins. The clinical landscape section evaluates current programs across different developmental phases, analyzing both successes and setbacks to identify key lessons for future development strategies. Our discussion of delivery systems addresses one of the field's most pressing challenges, exploring both molecular optimization strategies and advanced carrier technologies. Finally, we outline future directions encompassing molecular design optimization, E3 ligase toolbox expansion, organ‐specific targeting strategies, and approaches for overcoming clinical translation barriers. Through integration of mechanistic insights with clinical realities, this analysis provides both a current state assessment and a roadmap for future innovation as PROTAC technology transitions from a promising concept to transformative clinical reality across multiple therapeutic domains.

## 2 Design Principles and Degradation Mechanism of PROTACs

**FIGURE 1:**
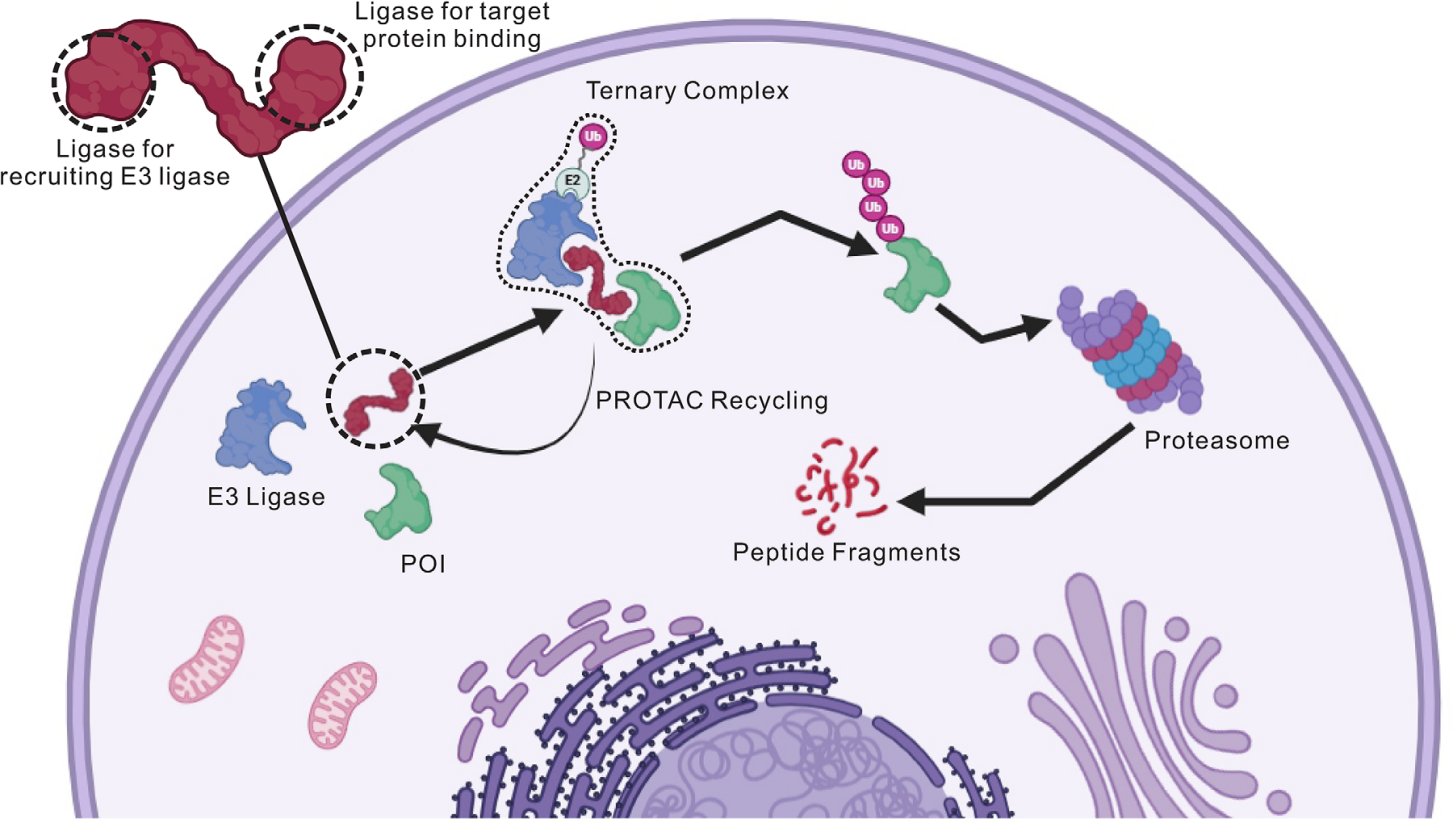
Mechanism and molecular architecture of PROTACs. Schematic illustration of a canonical PROTAC molecule comprising three components: a target protein (POI)‐binding ligand, an E3 ubiquitin ligase‐recruiting ligand, and a linker connecting the two functional domains. Degradation mechanism: The PROTAC facilitates ternary complex formation (POI–PROTAC–E3), enabling ubiquitin (Ub) transfer to lysine residues on the POI. The polyubiquitinated POI is subsequently recognized and degraded by the 26S proteasome. Following target degradation, the PROTAC molecule is recycled to engage additional target proteins, demonstrating the catalytic nature that enables sub‐stoichiometric target elimination.

PROTAC technology leverages the endogenous ubiquitin–proteasome system (UPS) to achieve selective elimination of target proteins and represents a hallmark of event‐driven pharmacology [29]. Unlike traditional occupancy‐based inhibition, PROTACs function by inducing spatial proximity between the POI and an E3 ligase, resulting in ubiquitination and subsequent degradation via the 26S proteasome [30, 31, 32]. Rather than functionally blocking the protein, this approach catalytically erases it from the system. A canonical PROTAC comprises three covalently linked components: a ligand that binds the POI, a ligand that recruits the E3 ligase, and a linker that bridges the two (Figure 1) [33]. The resulting chimeric molecule facilitates the formation of a POI–PROTAC–E3 ternary complex [34]. Upon productive ternary complex formation, the E3 ligase transfers ubiquitin moieties to lysine residues on the target protein, marking it for proteasomal degradation. The degradation efficiency, selectivity, and target scope of a PROTAC are influenced by several interdependent factors. While high‐affinity binding of both the POI ligand and the E3 ligand is important, the stability and cooperativity of the ternary complex are often more critical [35, 36, 37, 38, 39]. Studies have shown that even weak‐affinity ligands can drive potent degradation if the linker supports favorable ternary complex geometry [40, 41, 42]. Linker properties—such as length, flexibility, polarity, and spatial orientation—directly affect the protein–protein interface and determine whether the ternary complex adopts a ubiquitination‐competent conformation [43, 44, 45, 46]. Notably, the linker serves as a tunable element in PROTAC design, and its structural optimization has been shown to significantly impact both pharmacokinetics and target selectivity [47, 48]. Among E3 ligase ligands, CRBN‐ and VHL‐based molecules are the most widely used due to their defined structure–activity relationships, favorable stability, and synthetic accessibility. These ligands underpin the design of numerous PROTACs that have progressed to clinical evaluation. More recently, alternative E3 recruiters such as IAPs, MDM2, and DCAF family members have been explored to enhance tissue selectivity, reduce off‐target toxicity, and broaden therapeutic scope [49, 50, 51]. A unique advantage of PROTACs is their sub‐stoichiometric mode of action [52]. Once a target protein is degraded, the PROTAC molecule can be recycled, eliminating the need for continuous occupancy [53, 54]. This feature not only reduces systemic exposure requirements but also enables more robust activity against proteins harboring resistance mutations. Compared with classical inhibitors, PROTACs show clear superiority when targeting multidomain scaffolds, dynamic complexes, or proteins lacking conventional ligandable pockets [55]. PROTAC design is not merely a matter of chemical assembly—it is a systems‐level integration of conformational biology, complex thermodynamics, and pharmacological engineering. The linker, ternary complex dynamics, and degradation machinery must be harmonized to deliver efficient, selective, and therapeutically viable protein elimination.

## 3 Pharmacological Advantages of PROTACs Compared with Traditional Therapies

The emergence of PROTAC technology marks a paradigm shift in pharmacological strategy—from traditional inhibition to targeted degradation—opening new avenues in precision medicine. Table 1 systematically compares PROTACs with conventional therapeutic modalities across key dimensions, highlighting their unique advantages and clinical potential.

**TABLE 1:** Systematic comparison of PROTACs with conventional therapeutic modalities.

Feature/capability	Small molecule inhibitors	Monoclonal antibodies (mAbs)	Gene‐based medicines	PROTAC protein degraders
**Mechanism of action**				
Occupancy‐driven (binds & inhibits)	Yes	Yes	No	No
Event‐driven (catalytic degradation)	No	No	Possible	Yes
Sub‐stoichiometric activity (catalytic)	No	No	Possible	Yes
**Target engagement**				
Eliminates disease‐causing proteins	Indirectly	Indirectly	Yes	Yes
Disrupts Scaffolding/nonenzymatic function	Weak/no	Possible	Yes	Yes
Targets “undruggable” proteins	Rarely	Rarely	Yes	Yes
Targets intracellular proteins	Yes	No	Yes	Yes
Targets cell surface proteins	Possible	Yes	Limited	Possible
**Pharmacokinetics/administration**				
Broad tissue penetration	High	Low	Low/variable	Moderate
Systemic delivery	Yes	Yes	Limited/challenging	Yes
Oral bioavailability	High (many)	No	No	Possible/developing
Route of administration	Oral/Injection	Injection	Injection/complex	Oral/injection
**Development & practicality**				
Ease of manufacturing	Relatively easy	Complex	Very complex	Moderate/developing
Ease of development (drug discovery)	Challenging	Established	Complex	Emerging/potential
Potential for high dose dependence	Yes (often)	Yes	Variable	Low (catalytic)

### 3.1 Fundamental Differences in Mechanism of Action

#### 3.1.1 A Pharmacological Revolution: Occupancy‐Driven versus Event‐Driven

Traditional small‐molecule inhibitors and mAbs rely on an occupancy‐driven mechanism, requiring sustained binding to the target protein's active site to maintain inhibitory effects [40, 56, 57]. This necessitates high systemic drug concentrations, with effects diminishing as concentrations fall. In contrast, PROTACs utilize an event‐driven mechanism, inducing catalytic degradation of target proteins rather than merely inhibiting them, thereby achieving an irreversible elimination rather than reversible inhibition [58, 59, 60]. While genetic drugs may also offer event‐driven effects, their primary action is at the transcriptional level, limiting their ability to clear pre‐existing pathogenic protein aggregates.

#### 3.1.2 Sub‐Stoichiometric Activity: A Distinct Advantage

One of PROTACs’ most compelling features is their sub‐stoichiometric activity [61], where a small amount of PROTAC molecules can degrade large amounts of the target protein. This catalytic property allows a single PROTAC molecule to initiate multiple degradation cycles, in stark contrast to the 1:1 binding stoichiometry of conventional inhibitors and mAbs [3, 62]. While genetic therapies may demonstrate similar effects in regulating gene expression, they lack the proteome‐level catalytic degradation capability and often require complex delivery systems and delayed onset.

### 3.2 Target Engagement and Functional Disruption

#### 3.2.1 Direct Elimination versus Indirect Suppression of Pathogenic Proteins

Traditional small molecules and mAbs indirectly suppress pathogenic proteins by inhibiting their function, leaving the proteins intact and potentially active through alternative pathways [63, 64, 65]. In contrast, PROTACs and gene therapies eliminate pathogenic proteins—PROTACs through proteasomal degradation, and gene therapies by downregulating transcription—offering a more complete therapeutic strategy [66].

#### 3.2.2 Disruption of Scaffold and Nonenzymatic Functions

Many disease‐related proteins exert pathogenicity via scaffold functions or protein–protein interactions rather than enzymatic activity [67, 68, 69]. Traditional small molecules, designed to block catalytic sites, struggle in this context. mAbs are limited to extracellular targets. In contrast, PROTACs and genetic therapies can abrogate both enzymatic and nonenzymatic functions by eliminating the entire protein, making them particularly effective against structurally or functionally complex targets [70, 71, 72, 73].

#### 3.2.3 Unlocking the “Undruggable”

A major challenge in drug development is the vast array of so‐called “undruggable” targets—proteins lacking well‐defined binding pockets or catalytic sites [74, 75, 76]. PROTACs address this by requiring only a binding ligand to the target protein, without needing to inhibit a functional domain [77, 78]. Gene therapies indirectly regulate these proteins by modulating gene expression. Both approaches significantly expand the druggable proteome beyond traditional boundaries.

#### 3.2.4 Intracellular and Extracellular Target Coverage

Small molecules and PROTACs exhibit excellent cell permeability, enabling efficient targeting of intracellular proteins [79]. Gene therapies, though capable, often require elaborate delivery systems. mAbs are largely restricted to extracellular proteins due to their size [80, 81]. For membrane‐bound proteins, mAbs have inherent advantages; however, PROTACs—especially through innovative modalities such as antibody–drug conjugates (ADCs) or antibody–PROTAC conjugates (AOCs)—are increasingly bridging this gap [82, 83, 84, 85].

### 3.3 Pharmacokinetics and Administration

#### 3.3.1 Tissue Distribution

Small molecules offer superior tissue permeability, enabling broad distribution. PROTACs, with molecular weights between small molecules and biologics, offer moderate tissue penetration [86, 87, 88]. mAbs have limited distribution due to their size, while gene therapies vary widely depending on delivery systems and targeting strategies [89, 90].

#### 3.3.2 Delivery Platforms and Routes of Administration

Systemic delivery is feasible for all modalities but varies in complexity. Small molecules, mAbs, and PROTACs are generally straightforward to administer. Gene therapies face greater challenges due to their reliance on complex vectors [91]. Oral bioavailability is a critical consideration—small molecules lead in this area, while mAbs and gene therapies typically require parenteral routes. Although PROTACs face formulation challenges, advances in medicinal chemistry are improving their oral availability and diversifying their delivery options.

### 3.4 Development and Practical Considerations

#### 3.4.1 Manufacturing Complexity

Small molecules are synthetically straightforward. mAbs require biotechnological production [92], while gene therapies involve vector construction and packaging, making them the most complex [93, 94]. PROTACs occupy an intermediate position—more complex than small molecules, but simpler than biologics or gene therapies.

#### 3.4.2 Drug Discovery Efficiency

Small molecule development is hindered by the limitations of target druggability [95]. mAbs benefit from mature development pipelines. Gene therapies are technically challenging due to multidisciplinary requirements [96]. PROTACs, as an emerging modality, show rapid development potential and are increasingly supported by structure‐based drug design and high‐throughput screening.

#### 3.4.3 Dose Dependency and Therapeutic Window

Traditional therapies often show strong dose dependence, requiring high systemic exposure for efficacy. Gene therapies vary in dose response [97]. PROTACs, due to their catalytic nature, display lower dose dependency and wider therapeutic windows, potentially reducing toxicity and side effects. A systematic comparison of PROTACs with traditional modalities clearly underscores the transformative value of TPD in modern therapeutics. PROTACs provide mechanistic innovations, such as event‐driven action and sub‐stoichiometric efficiency, pharmacological breadth, including engagement with undruggable targets and disruption of nonenzymatic functions, and practical advantages like lower dose dependency and potential for oral delivery. Nevertheless, challenges remain, including optimizing tissue permeability, improving oral bioavailability, and reducing manufacturing costs. With ongoing technological advancements and accumulating clinical experience, PROTACs are poised to play a central role in future therapeutic paradigms, offering safer, more effective, and convenient treatment options for a wide range of diseases.

## 4 Representative Targets and PROTAC Molecules

**FIGURE 2:**
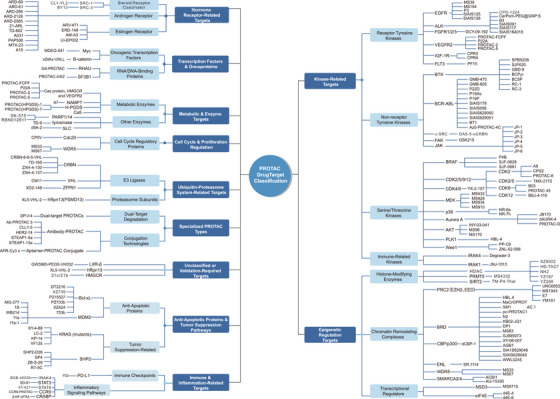
Therapeutic targets and representative PROTAC degraders. The major target classes addressed by PROTAC technology include kinases, transcription factors, hormone receptors, epigenetic regulators, immune targets, metabolic enzymes, and components of the ubiquitin–proteasome system. Key representative degraders are illustrated for each target category.

PROTAC technology has exploded onto the drug discovery scene in recent years, capturing attention primarily because it works so differently from traditional inhibitors. While conventional drugs need to continuously occupy their targets to maintain effect, PROTACs can essentially “tag” proteins for destruction and walk away [98]. This makes them particularly attractive for going after those notorious “undruggable” proteins that have frustrated medicinal chemists for decades. Recent breakthroughs in molecular engineering, E3 ligase recruitment strategies, structural optimization, and getting these molecules into cells have pushed multiple PROTAC candidates into clinical trials, opening up therapeutic possibilities that seemed impossible just a few years ago (Figure 2).

### 4.1 Kinase Targets

Kinases have emerged as the poster children of PROTAC development, and for good reason. These proteins occupy central roles in numerous disease pathways, and an extensive library of kinase inhibitors is already available to serve as starting points for PROTAC design—providing a significant advantage. Kinases span several major families, including receptor tyrosine kinases (EGFR, FGFR, VEGFR, IGF‐1R, FLT3) [99], nonreceptor tyrosine kinases (Bruton's tyrosine kinase [BTK], BCR–ABL, SRC, FAK, JAK) [100], serine/threonine kinases (CDK, MEK, BRAF, AKT, PLK1, Wee1, p38, Aurora A/B) [101], and immune‐related kinases (interleukin‐1 receptor‐associated kinase [IRAK]1/4) (Figure 2) [72, 102]. Take EGFR—it has become something of a showcase for what PROTACs can accomplish. Molecules such as MS154, MS39, SIAIS125, and SIAIS126 have demonstrated the ability to selectively degrade mutant EGFR variants (L858R, T790M) [103, 104, 105], while leaving the normal protein alone, something that has been a holy grail in lung cancer research. But the EGFR story does not stop there. Researchers have gotten creative with delivery approaches, like Cer–Pom–PEG@GNP‐S [106], which packages the degrader in nanoparticles for better cellular uptake. The SIAIS team has been particularly productive, churning out SIAIS091 [107], SIAIS117 [108], and SIAIS164018 [109], each iteration getting better at hitting the target. PROTAC–CPF brings fresh chemistry to the table with novel E3 ligase recruitment strategies. FGFR has not been left behind either—DGY‐09‐192 [110, 111], built around the BGJ398 [112, 113, 114] inhibitor backbone, can efficiently degrade FGFR1/2 and shut down their downstream signaling. For VEGFR2, successful inhibition of angiogenesis has been achieved with molecules such as P22A, PROTAC‐2, and PROTAC‐5, with PROTAC‐5 demonstrating particularly impressive efficacy in disrupting blood vessel formation [115, 116, 117]. Similar progress has also been made in targeting IGF‐1R [118] and FLT3 [119] have their champions too—CPR3/CPR4 for breast cancer applications and PF15 for leukemia, respectively. The FLT3 field has really taken off recently with SPBS208, SJF020, GBD‐9, and BCPyr all bringing different structural approaches to tackling acute myeloid leukemia. Moving to the nonreceptor kinases, BTK was actually one of the early PROTAC success stories. GMB‐475 [120], RC‐1 [121], and RC‐3 [122] paved the way, but the field has matured considerably. GMB‐805 improved on GMB‐475's pharmacokinetics, while P22D explored completely different chemical scaffolds [123]. P19As and P19P [124] represent newer thinking about how to achieve selectivity. All of these work by hijacking either CRBN or VHL E3 ligases to tag BTK for destruction, which has been particularly exciting for B‐cell malignancies [125, 126]. BCR–ABL [124, 127] is where PROTACs have really shown their muscle against drug resistance. This fusion protein is notorious for developing resistance mutations to kinase inhibitors, but degraders like SIAIS178 [128], BT1 [129], and AZO–PROTAC‐4C [130] can still take it out even when it is mutated. The SIAIS team has been busy here too—SIAIS056 [131] was an early proof‐of‐concept, while SIAIS629050 and SIAIS629051 [132, 133] represent the next generation with improved selectivity profiles. It is encouraging to see chemistry solving problems that seemed intractable. SRC and FAK have had their moments too, with CL1‐YL2 making headway against SRC signaling and FAK degraders showing promise for blocking cancer cell migration [134, 135]. But JAK is where things get interesting from a scope perspective. What started with just GSK215 [136] has exploded into the JP series—JP‐1 through JP‐6—representing a systematic effort to optimize JAK degradation. JP‐1 got the ball rolling with JAK1/2 targeting, JP‐2 sharpened the selectivity, JP‐3 tackled cell penetration issues, JP‐4 focused on JAK2 specificity, JP‐5 proved the concept in inflammatory disease models, and JP‐6 finally achieved the pharmacokinetic properties needed for clinical development. The serine/threonine kinases tell a similar story of rapid expansion. CDKs control cell division, so naturally they have attracted intense interest. Early molecules like YKL‐06‐101 [137], BSJ‐4‐116 [138], and L055 [139] proved the concept in leukemia and breast cancer models, but the field has exploded from there. A9 emerged as a particularly potent CDK4/6 degrader [140], CPS2 brought cell cycle specificity, PROTAC‐8 went for a multitarget approach hitting CDK2/4/6 simultaneously [141], and TMX‐2172 [142] explored new linker chemistry. Then you have CDK2/5 for dual targeting, CDK9 specifically for transcriptional control, PRN showing impressive cancer cell activity, and PROTAC 45 with enhanced stability. It has been a rapid evolution. MEK sits at a critical node in cancer signaling, so the MS series (MS432, MS928, MS910, MS934) along with YX‐2‐107 [143] have been attacking it from multiple angles. MS934 represents the latest thinking in the MS series [144], while YX‐2‐107 brings an alternative chemical approach. BRAF [145], particularly the V600E mutant that drives many melanomas, has been targeted by P4B, SJF‐0628, and the newer SJF‐0651, which shows improved selectivity for the mutant form. AKT pathway degradation has been another success story. INY‐03‐041 [146, 147] can take out all three AKT isoforms, which is impressive given how central this pathway is to cancer cell survival. MS98 offers AKT1 selectivity when you need it, while MS170 provides pan‐AKT coverage [148]. The fact that these molecules can improve both signal persistence and antiproliferative effects suggests they're doing something fundamentally different from inhibitors. Cell cycle control is another arena where PROTACs are making their mark. PLK1, Wee1, and Aurora kinases all play critical roles in mitosis, making them attractive cancer targets. HBL‐4 (PLK1) [149], PP‐C8 [150] and ZNL‐02‐096 (Wee1) [151], and JB170 [152], PROTAC‐D, and dAURK‐1 (Aurora) [153, 154] have all shown they can trigger G2/M arrest and tumor suppression. ZNL‐02‐096 has been particularly interesting for DNA damage response studies, while dAURK‐1's Aurora A specificity makes it useful for dissecting mitotic mechanisms. Even p38, with its role in inflammation and immune regulation, has its degraders—NR‐6a and NR‐7 h can knock down p38α/β at nanomolar concentrations [133, 155]. And the immune kinases IRAK1/4 have not been forgotten, with Degrader‐3 and INJ‐1013 offering new ways to modulate Toll‐like receptor and NF‐κB signaling. INJ‐1013's IRAK1 selectivity is particularly valuable for inflammatory disease research [156, 157].

What is remarkable about this kinase PROTAC explosion is how it reflects the underlying biology. These are not just random targets—they represent the key nodes in disease‐relevant signaling networks. The rapid expansion from initial proof‐of‐concept molecules to numerous validated degraders across nearly every major kinase family underscores the broad applicability of this technology. More importantly, it is giving us shots at targets that traditional drug discovery could not touch, which could translate into new therapeutic options for patients who have run out of alternatives.

### 4.2 Transcription Factors

Transcription factors, functioning within the cell nucleus and predominantly featuring flat surface structures lacking druggable pockets, have long been considered “undruggable” targets for traditional small molecules and mAbs [158, 159]. PROTAC technology provides a novel intervention modality for such targets (Figure 2). Myc, a representative oncogenic transcription factor, serves as a core driver for maintaining tumor cell metabolism and cell cycle progression [160]. Its basic helix–loop–helix–zipper structure exists in a disordered state when not bound to its partner protein MAX. MDEG‐541 [161] represents a Myc‐targeting PROTAC molecule that, through linking Myc ligands with VHL ligands and optimizing linker configuration, can induce formation of tight Myc–POI and E3 complexes, ultimately achieving targeted intracellular degradation and effectively inhibiting Myc target gene transcription.

β‐Catenin, as the core transcriptional regulatory factor of the Wnt/β‐catenin signaling pathway, is abnormally activated in various malignancies including colorectal cancer [162] and hepatocellular carcinoma [163]. xStAx–VHLL [164] represents a PROTAC molecule designed for β‐catenin degradation, with its design strategy stemming from identification of small molecule‐bindable “receptor arms” within the β‐catenin structure, while utilizing VHL ligands to guide E3 binding through regulation of linker flexibility and spatial conformation to enhance complex formation probability. In functional validation, xStAx–VHLL effectively reduces intracellular β‐catenin protein levels, thereby disrupting its transcriptional complex formation with TCF/LEF.

RNA/DNA‐binding proteins also represent important transcriptional regulatory nodes, with multiple related targets currently incorporated into PROTAC development pipelines. RHAU (DHX36)[165], a G‐quadruplex structure‐recognizing RNA helicase, plays important roles in regulating oncogene mRNA stability. G4–PROTAC represents the first degrader targeting G‐quadruplex structures, successfully inducing G‐quadruplex degradation through recognition of G4 binding sites for ligand construction and coupling with CRBN ligands, thereby reducing related mRNA stability and translation efficiency. SF3B1, as a core component of the spliceosome U2 complex, plays crucial roles in pre‐mRNA splicing [166]. PROTAC–O4I2, constructed based on coupling of 2‐aminothiazole derivative O4I2 with thalidomide, can specifically degrade SF3B1 with an IC_50_ of 0.244 µM. This molecule induces SF3B1 degradation through CRBN‐dependent mechanisms, leading to splicing errors and transcriptional program disorders, subsequently inducing cell apoptosis. In Drosophila intestinal tumor models, PROTAC–O4I2 significantly improves survival rates and interferes with tumor stem cell maintenance and proliferation [167].

The antagonistic roles of transcription factors STAT3 and STAT5 signaling pathways in dendritic cells represent important factors determining immunotherapy responsiveness. Research by Zhou et al. [168] not only revealed the critical role of STAT3 in suppressing DC function and limiting T‐cell activation within the tumor microenvironment but also developed highly efficient STAT3‐targeting PROTAC molecules—SD‐36 and SD‐2301. These molecules achieve selective STAT3 degradation through “event‐driven” mechanisms, thereby relieving its suppression of DCs, enhancing antigen presentation capabilities and T‐cell immune activity. In various tumor models, these STAT3 degraders significantly inhibited tumor progression, particularly demonstrating significant synergistic effects in immune checkpoint therapy‐resistant models, showing promising safety profiles and clinical translation potential.

PROTAC research targeting transcription factors and their related regulatory factors has gradually overcome “undruggable” limitations, with current strategies primarily focusing on indirect interference (such as the BRD4–cMyc axis), structure‐induced binding (such as β‐catenin), and precise degradation of posttranscriptional regulatory targets (such as RHAU and SF3B1) [169]. With advances in protein–ligand structural analysis and linker conformational engineering, more traditionally undruggable transcriptional regulatory factors will be incorporated into targeted degradation paradigms, expanding the target boundaries for precision anticancer therapy.

### 4.3 Hormone Receptors

Among hormone receptor‐related targets, AR and estrogen receptor (ER) represent among the most mature targets for PROTAC technology, with multiple representative degraders emerging (Figure 2). The first PROTAC drug to enter clinical trials, ARV‐110 [170], targets prostate cancer treatment. AR, a nuclear receptor family member, is persistently activated in malignancies such as prostate cancer. Multiple PROTACs have been developed, including ARD‐69 [47], ARD‐61 [171], ARD‐266 [172], ARD‐2128 [173], ARD‐2585 [174], 21‐ARL, TD‐802 [175], A031, PAP508, MTX‐23, A16, and VPC‐14228 derivatives (Figure 2) [176]. The ARD series (such as ARD‐69, ARD‐2128) are predominantly derived from Pfizer AR antagonists, forming highly efficient ternary complexes through linkage with VHL or CRBN ligands, capable of inducing sub‐nanomolar AR degradation in AR‐positive cells such as LNCaP and VCaP [47, 177]. MTX‐23 [178] further addresses both full‐length AR and splice variant AR‐V7, which is particularly significant for patients with hormone blockade therapy resistance [179]. PAP508 and VPC‐14228 expand the targeting spectrum through different binding sites, demonstrating excellent cellular inhibition and tumorigenesis suppression capabilities.

ERα is abnormally activated in over 70% of breast cancers [180]. Representative molecule ARV‐471 has entered Phase III clinical trials [181], demonstrating promising oral pharmacokinetics and degradation activity. Its degradation mechanism depends on highly efficient conjugation of ERα ligands with CRBN. Beyond ARV‐471, molecules such as I‐6, ERD‐148, AM‐A3, and UI‐EP002 have been developed [182, 183, 184, 185]. For instance, ERD‐148 demonstrates low nanomolar DC_50_ values against both MCF‐7 and ERα mutants (Y537S/D538G), while AM‐A3 and its improved versions exhibit maximum degradation rates exceeding 90% [186]. UI‐EP002 represents a unique pan‐ER degrader capable of targeting not only ERα but also ERβ and GPER, showing dual regulatory potential for membrane and nuclear receptors.

PROTAC design strategies for AR/ER targets primarily focus on two aspects: first, selecting existing antagonists (such as enzalutamide, tamoxifen) [187] as recognition arms; second, pairing with optimized E3 linkers (VHL, CRBN, or DCAF11) [188], regulating stereochemical conformation and proximity through linker optimization. These molecules generally demonstrate sustained degradation effects far superior to traditional inhibitors and are most advanced in clinical translation, representing important new directions for hormone‐driven tumor therapy.

### 4.4 Antiapoptotic and Oncogenic Proteins

Among “undruggable” targets, antiapoptotic proteins and oncogenic mutant proteins have long been considered difficult to effectively inhibit. However, with PROTAC technology development, this situation is rapidly changing. Degraders targeting key proteins such as Bcl‐2 family members, MDM2, KRAS, and SHP2 have successively emerged, demonstrating considerable selectivity and biological activity (Figure 2).

Bcl‐xL, a key regulatory factor inhibiting cell apoptosis within the Bcl‐2 family, is widely involved in cell survival and chemotherapy resistance processes in various malignancies [189]. Traditional inhibitors such as navitoclax, while showing some efficacy, are limited in clinical application due to platelet toxicity [190]. To address this challenge, multiple Bcl‐xL inhibitor‐based PROTACs have emerged, including DT2216, X2739, P215527, PZ703b, XZ424, and 753b [191, 192, 193, 194, 195]. These molecules predominantly use ABT‐263 or its derivatives as POI ligands, combined with CRBN or VHL recruitment elements, significantly reducing platelet toxicity burden by degrading Bcl‐xL protein rather than merely inhibiting its function. DT2216 [196, 197], in particular, has entered preclinical research stages, demonstrating promising safety and selectivity in various Bcl‐xL‐dependent tumor models, providing new approaches for addressing Bcl‐xL “druggability challenges.”

Similar to Bcl‐xL, MDM2, as an E3 ligase for p53 protein, represents a core node for negative p53 regulation. In TP53 wild‐type tumors, MDM2 overexpression can mediate p53 degradation through ubiquitination, suppressing its tumor suppressor function [198]. Multiple MDM2‐targeting PROTAC molecules have been reported, such as MG‐277, WB214, 1B, 11a, and 11a‐1, all designed using Nutlin‐class structures as templates [199, 200, 201]. These molecules demonstrate stronger antiproliferative capabilities and broader applicability compared with traditional MDM2 inhibitors by restoring p53 stability and activating its downstream apoptotic pathways, particularly showing potential in p53‐dependent tumors such as malignant melanoma and soft tissue sarcomas.

For mutant protein targets, BRAFV600E and KRASG12C represent typical driver mutations long considered “undruggable” [202]. BRAFV600E‐related degraders such as SJF‐0628 [203] and P4B [204], designed based on vemurafenib [205] and BI882370, respectively, retain selectivity in wild‐type BRAF backgrounds while significantly inhibiting MEK/ERK pathway activity. SJF‐0628 achieves >95% *D*_max_ degradation efficiency, while P4B effectively inhibits tumor cell proliferation, demonstrating mutation‐specific effects. KRASG12C, due to its smooth surface structure and lack of binding cavities, has been considered a representative “undruggable” target [206]. With the emergence of covalent inhibitor MRTX849 [207], PROTAC LC‐2 [208] achieves highly efficient KRASG12C degradation through coupling with VHL ligands. Subsequently developed molecules KP‐14, YF135, and XY‐4‐88 demonstrate anticlonogenic formation and signal blocking effects in various KRAS mutation models [209, 210]. YF135 represents the first reversible covalent PROTAC, providing new molecular tools for overcoming drug resistance risks caused by covalent binding limitations.

SHP2, a protein tyrosine phosphatase, plays critical signal bridging and amplification roles across multiple receptor tyrosine kinase pathways, representing a key synergistic factor for EGFR, ALK, and other driver mutation tumors [198]. Conventional SHP2 inhibitors exhibit limited efficacy due to binding site constraints, whereas next‐generation SHP2 PROTACs have reshaped the pharmacological landscape through whole‐protein degradation strategies. To achieve selective SHP2 degradation, several PROTAC molecules targeting SHP2 have been developed: SHP2‐D26, constructed from SHP099 and VHL ligands, induces rapid and sustained SHP2 degradation at nanomolar concentrations while significantly inhibiting downstream ERK activation [199]; SP4 employs CRBN ligands conjugated to SHP099 [200], optimizing conformational pairing through PEG linkers to achieve approximately 40% SHP2 degradation in HeLa cells with modest antiproliferative effects; ZB‐S‐29 utilizes TNO155 as the binding moiety, also based on the CRBN system [201], achieving >90% SHP2 protein degradation in MV‐4‐11 cells. These molecules comprehensively block SHP2‐dependent signal transduction, thereby enhancing synergistic efficacy with RTK inhibitors, particularly demonstrating promising applications in lung cancer, breast cancer, and other malignancies [202]. Of particular importance, SHP2 function within the RAS–ERK pathway is essential for maintaining sustained tumor cell proliferation and immunosuppressive states [203]. Further investigations reveal that ZB‐S‐29 treatment results in cell cycle arrest and apoptosis induction [204]. Additionally, the R1‐5C molecule combines RMC‐4550 [205] with pomalidomide, demonstrating superior selective degradation capabilities and effectively attenuating MAPK pathway signal transduction while inhibiting tumor cell growth.

Overall, targeted degradation strategies for antiapoptotic and tumor suppressor pathway‐related proteins are breaking through inherent “undruggable” barriers. The common characteristics of this target class include structural complexity, functional diversity, and high involvement in tumor cell survival regulation. PROTACs achieve novel dimensional intervention modalities by reconstructing protein fate, not only providing breakthroughs for treating highly drug‐resistant malignancies with cryptic targets but also continuously expanding the theoretical and practical boundaries of protein degradation technology.

### 4.5 Epigenetic Regulatory Targets

Among epigenetic regulatory targets, BRD4 undoubtedly remains one of the most intensively studied subjects [211]. As a core member of the BET family, BRD4 can directly recruit transcriptional initiation complexes, activating expression of multiple oncogenes including Myc. The field has seen an explosion of creativity in BRD4 degrader design, moving well beyond the early proof‐of‐concept molecules. Early developed PROTAC molecules such as dBET1 [212], MZ1 [213], and ARV‐825 [214] have been widely used to validate their degradability and antitumor potential. dBET1 uses pomalidomide as a CRBN ligand and represents among the first molecules to achieve highly efficient BRD4 degradation, effectively downregulating Myc expression and inducing acute myeloid leukemia cell apoptosis. MZ1 employs the VHL system, enhancing ternary complex stability through optimized linker conformation, demonstrating excellent degradation efficiency in various solid tumor models. ARV‐825 integrates BRD4 ligand OTX015 with pomalidomide, combining broad‐spectrum activity with favorable pharmacokinetic properties [215]. However, it should be noted that ARV‐825 is actually a pan‐BET protein degrader that can simultaneously degrade multiple members of the entire BET protein family. In addition to BRD4, BRD2, BRD3, and others can also be degraded, although the degradation effect on BRD4 is the most pronounced. This multitarget characteristic is determined by the molecular design of ARV‐825, as its BRD4‐binding domain inherently has affinity for multiple BET proteins, thus the final PROTAC molecule also retains this multitarget feature.

Researchers have continued to push the boundaries with increasingly sophisticated designs. QCA represents a newer generation of BRD4 degraders that builds on lessons learned from earlier molecules, offering improved potency profiles [216]. The field took another leap forward with SJ995973 [217], which utilizes phenyl‐glutarimide analogs to achieve remarkable stability while maintaining potent BRD4 degradation at picomolar concentrations—a real game‐changer for in vivo applications. Meanwhile, molecules like DB1, VS3, and XY‐06‐007 [218] have explored different chemical scaffolds and linker strategies, each bringing something unique to the table in terms of selectivity profiles or pharmacokinetic properties. What is particularly exciting is how these diverse approaches have collectively expanded our understanding of what makes an effective BRD4 degrader. Beyond BRD4, multiple chromatin regulatory proteins outside the BET family have gradually entered PROTAC development perspectives. Taking PRC2 as an example, its core members EZH2 and EED synergistically regulate H3K27me3 silencing marks, serving as oncogenic drivers in various lymphomas and solid tumors [219, 220]. Degrader series such as HBL‐4, MaCrOPROTAC.1 [221], SM1, pc‐PROTAC1 [222], and N2 have achieved effective targeting of PRC2 complexes, with some molecules retaining degradation capabilities even in EZH2 inhibitor‐resistant backgrounds, expanding their clinical applicability. HDAC3, PRMT5, and SIRT2 have also been gradually incorporated into targeting spectra, mediated by molecules such as UNC6852 [223], MS1943 [224], E7 [225], and YM181 [226], respectively, demonstrating multidimensional intervention potential for histone acetylation, arginine methylation, and deacetylation balance. Another key target class is CBP/p300, histone acetyltransferases that regulate transcriptional initiation, which can be selectively degraded through molecules such as dCBP‐1 [227, 228]. The CBP/p300 degrader landscape has really matured over the past few years, with researchers developing increasingly sophisticated molecules. Beyond dCBP‐1, AGB1 provides an alternative chemical approach to CBP targeting [229]. The SIAIS team has been particularly productive here, developing SIAIS629048 and SIAIS629049 as part of their systematic exploration of CBP/p300 degradation—these molecules showcase how iterative medicinal chemistry can fine‐tune both potency and selectivity. The compound WWLJ245 rounds out this collection with yet another structural approach, demonstrating that there are multiple viable paths to effective CBP/p300 degradation. Compared with traditional inhibitors, these degraders potentially circumvent toxicity accumulation issues while maintaining dynamic regulation of epigenetic states and improving therapeutic windows.

Additionally, the compounds, ENL [230], WDR5 [231], and SMARCA2/4 [232, 233] structural proteins have also become recent focal points. ENL promotes abnormal activation of leukemic transcriptional programs through binding to transcriptional elongation complexes, with selective degrader SR‐1114 effectively interrupting this process [234]. WDR5 degraders MS33 and MS67 show promise for MYC‐driven tumor models [235, 236]. SMARCA2/4 belong to the SWI/SNF complex, and in SMARCA4‐deficient backgrounds, SMARCA2 becomes a critical “synthetic lethal” dependency target, with related degraders such as ACBI1 and AU‐15330 demonstrating promising targeting and therapeutic potential in SMARCA4‐deficient and NSCLC models [237]. At the transcriptional regulation level, NSD3 and eIF4E as emerging epigenetic targets have also received attention. NSD3 degrader MS9715 can effectively disrupt chromatin open state maintenance, while eIF4E‐targeting degraders d4E‐4 and d4E‐6 attempt to sever cancer cell mRNA translation activity through protein degradation strategies, thereby suppressing oncogenic protein expression from the source [238, 239]. Although these targets remain in validation or optimization stages, they possess breakthrough value in target structure, regulatory levels, and therapeutic indication selection.

### 4.6 Immune and Inflammatory Targets

Among immune and inflammatory targets, the PD‐1/PD‐L1 pathway and STAT3 represent extremely promising key nodes in current PROTAC research (Figure 2). PD‐L1, through binding with PD‐1 on T‐cell surfaces, inhibits their activation, maintaining immunosuppressive states within tumor microenvironments [240]. While traditional antibody drugs have achieved clinical progress, their therapeutic effects remain limited due to individual efficacy variations, toxicity accumulation, and secondary resistance issues. PROTAC technology provides novel strategies for overcoming these challenges. Several molecules capable of inducing PD‐L1 degradation have been developed. Small molecule degrader P22 can mediate CRBN‐dependent degradation of PD‐L1 protein, demonstrating strong immune activation capabilities [241]. Additionally, strategies for indirectly inducing PD‐L1 cleavage and inactivation through interference with its palmitoylation modifications have also progressed. Cyclic peptide PROTACs can target transferase DHHC3 [242, 243], reducing PD‐L1 stability in tumor cells and promoting IFN‐γ and TNF‐α secretion, reshaping immune environments from the modification level. In contrast, PD‐1, due to its transmembrane structure lacking designable binding sites, has not been effectively degraded through traditional small molecule PROTACs. Recently, a study employing peptide‐structured peptide–PROTACs achieved the first successful intracellular PD‐1 protein degradation [244], resolving the technical bottleneck of this target being “undegradable.” This strategy integrates cell‐penetrating peptides, target protein recognition peptides, linkers, and VHL recruitment peptides into a unified system. The designed peptide PROTAC can significantly reduce PD‐1 protein expression at concentrations below 5 µM in cervical cancer cells C33A and HeLa, functioning through proteasome‐dependent pathways. Functional experiments confirm its ability to significantly weaken tumor cell suppression of T‐cells, enhance immune responses, and demonstrate synergistic tumor proliferation inhibition effects when combined with cisplatin. This research not only validates that PD‐1 protein can be targeted for degradation through PROTAC pathways but also demonstrates the unique advantages of peptide molecules in handling transmembrane proteins. In upstream regulation of the PD‐1/PD‐L1 pathway, STAT3, as a key transcription factor, regulates expression of immunosuppressive factors such as PD‐L1 and IL‐10, playing important roles in immune escape and tumor progression. Multiple STAT3 PROTACs have achieved effective degradation, such as SD‐91 [245] and KP‐18, both utilizing STAT3 inhibitors as ligand constructs. Postdegradation, they can significantly inhibit STAT3 target gene expression and tumor cell proliferation, possessing broad‐spectrum immunoregulatory capabilities.

Additionally, some immune‐related factors have also developed effective degradation tools. Macrophage migration inhibitory factor participates in inflammatory factor secretion and tumor immunosuppression formation, with its degradation capable of blocking proinflammatory signaling axes [246]. CCR9, a chemokine receptor involved in lymphocyte recruitment, has been targeted by molecules such as CCRB–PROTAC, which demonstrate regulatory potential in mucosa‐associated tumors and autoimmune disease models. CRABP (cellular retinoic acid‐binding protein) plays important roles in regulating retinoic acid metabolism and nuclear receptor activity, with related degrader β‐NF–ATRA validated in retinoic acid pathway‐dependent disease models [247, 248].

PROTAC designs targeting immune and inflammatory regulatory proteins continuously advance the possibilities for targeted degradation of transmembrane proteins, transcription factors, and immune auxiliary molecules. Particularly, PD‐1 peptide PROTAC strategies demonstrate clear advantages, providing new intervention pathways for overcoming immune checkpoint blockade therapy resistance and enhancing T‐cell activation levels, while establishing molecular foundations for future combination therapeutic strategies.

### 4.7 Metabolic and Enzymatic Targets

Among metabolic and enzymatic targets, NAMPT, the rate‐limiting enzyme in NAD⁺ biosynthetic pathways, frequently participates in dynamic physiological regulation and stress responses (Figure 2). Inhibiting MDSC growth and boosting antitumor immunological responses, PROTAC A7, created by linking VHL ligands to NAMPT inhibitors, destroys cellular NAMPT at small nanomolar concentrations while lowering external NAMPT levels [249, 250]. Its structurally optimized version B3 demonstrates stronger activity in A2780 cells (DC50 < 0.17 nM, *D*_max_ > 90%) [251].

H‐PGDS plays a critical role in prostaglandin D_2_ (PGD_2_) synthesis and represents an important therapeutic target in inflammatory and allergic conditions. Significant progress has been made in this field with the development of molecules targeting this enzyme through various mechanisms. Among these molecules, PROTAC(HPGDS)‐7 has demonstrated notable efficacy in reducing H‐PGDS protein levels and suppressing PGD_2_ production [252, 253]. This molecule, along with related degraders in this space, provides new molecular tools for regulating inflammatory mediator synthesis. Mitochondrial carbonic anhydrase Ca5 participates in cellular pH balance and metabolic regulation, with preliminary studies constructing PROTAC molecules targeting it, indicating that its protein stability can be modulated through degradation mechanisms.

Among other enzyme classes, PARP1/14 degradation mechanisms can achieve complete clearance of DNA repair enzymes, helping resolve residual activity and drug resistance issues faced by traditional PARP inhibitors in tumor therapy [254]. An interesting aspect of the PARP1/14 degradation approach is its potential to overcome the limitations associated with conventional PARP inhibitors, particularly in addressing resistance mechanisms. Tyrosinase plays roles in melanin synthesis and related skin lesions, with TD‐9 representing one of its early PROTAC degraders, providing structural foundations for melanin metabolism regulation [255]. SLC (solute carrier) family members, as transmembrane transport proteins, are difficult to effectively modulate with traditional drugs due to their strong hydrophobicity and complex transmembrane structures [256]. d9A‐2 [257], developed using AP1867 ligands, achieves targeted degradation of SLC proteins, providing feasible solutions for modulating membrane transport functions through protein degradation methods. Additionally, molecules such as P22A, PROTAC‐2, and PROTAC‐5 can simultaneously degrade multiple proteins related to cellular signaling and metabolism, including Cas proteins, HMGCR, and VEGFR2. PROTAC–FCPF also possesses multitarget binding capabilities, with configurations supporting synchronized intervention of key enzymes during cellular proliferation and metabolic regulation processes.

### 4.8 UPS‐Related Targets

In UPS‐related targets, researchers focus not only on substrate protein degradation but also begin exploring targeted modulation of key regulatory factors within the system. For instance, E3 ligases themselves can become targets for PROTAC molecules. CRBN and VHL represent the most commonly used E3 ligands, while degradation attempts of these components themselves expand PROTAC application boundaries in “self‐regulation” and systemic intervention aspects (Figure 2). Representative molecules such as CRBN‐6‐5‐5–VHL, TD‐165, ZXH‐4‐130, and ZXH‐4‐137 can regulate substrate recognition characteristics by inducing CRBN self‐degradation, while CM11 targets VHL for feedback regulation [258, 259, 260]. XD2‐149 can effectively downregulate ZFP91 expression. ZFP91, as an E3 ubiquitin ligase, has been implicated in the regulation of inflammatory and stress response pathways, including potential connections to NF‐κB signaling, showing prospects for inflammation and cancer intervention [261].

Additionally, PSMD13 (also known as hRpn13), as a 26S proteasome regulatory subunit, has degrader XL5–VHL‐2 proven to block IκBα degradation and inhibit NF‐κB activation [262], bringing new strategies for inflammatory disease treatment. Regarding cell cycle regulation‐related targets, Cdc20 serves as a core regulatory factor driving mitotic progression, with degrader CP5V specifically inducing its rapid ubiquitination and interfering with cell cycle progression [263]. WDR5, as a core subunit of the MLL/SET1 methyltransferase complexes, regulates histone H3K4 methylation and gene expression [264]. PROTAC‐mediated WDR5 degradation has shown therapeutic potential in various cancer models by disrupting oncogenic transcriptional programs.

Simultaneously, some uncategorized targets have also been validated for degradability in recent years. LXR‐β, a nuclear receptor participating in cholesterol homeostasis and immune regulation [265], has degrader GW3965–PEG5–VH032 effectively interfering with its transcriptional functions and demonstrating effects in atherosclerosis models [266]. Research on these “nontraditional” targets continuously pushes the boundaries of PROTAC target space expansion, suggesting the technology's potential for modulating complex signaling axes and metabolic networks.

These targets, located at ubiquitin system hub nodes or cell cycle core regulatory positions, provide important breakthroughs for precisely regulating intracellular homeostasis and developing novel anticancer or anti‐inflammatory therapeutic methods. Unlike traditional POI targeting strategies, PROTACs can achieve multipathway synergistic regulation through “degrading core regulatory factors.”

### 4.9 Novel PROTAC Formats and Conjugation Technologies

Several new PROTAC formats have been developed due to the development of PROTAC technology (Figure 2), including dual‐target degradation and conjugation technologies, which represent novel PROTAC design methodologies with several innovations. DP‐V‐4 [267] represents dual‐target‐specific degradation technologies capable of simultaneously degrading two protein targets, EGFR and PARP. Through antibody targeting, Ab–PROTAC3 uses antibody–PROTAC conjugation techniques to enhance PROTAC‐specific delivery. For specific targeting, aptamer–PROTAC conjugates (APCs) use the high‐specificity binding abilities of aptamers. These fascinating PROTACs expand the range of targets that degrader systems can handle.

Specific types are still growing in the PROTAC research. Progressive additions to degradation approaches include traditional driver proteins, E3 ligases, proteasome proteins, and governmental cells [268]. Such targets are typically found at upstream or main nodes of protein homeostasis regulation, where degradation frequently instantly affects many signaling axes, leading to enhanced intervention effects. Most modern PROTACs still primarily rely on VHL and CRBN E3 ligases to obtain substrate ubiquitination, with their reasonably sophisticated and stable derived receptor designs making them chosen choices for degradation system construction despite the increase in target diversity. Newly developed E3 enzyme targets, like ZFP91 [261], provide stronger organized regulatory possibilities, demonstrating large therapeutic prospects in neurotic states, including tumors, disease, and cell cycle abnormalities.

### 4.10 Summary and Comparative Analysis of Representative PROTAC Targets

PROTAC has advanced rapidly across multiple classes of targets, including kinases, transcription factors, hormone receptors, and others [269]. In selecting a suitable target, several principles must be considered: disease context, with oncology remaining the dominant area but opportunities expanding in immunology, neurology, and metabolic disease; structural accessibility and the availability of high‐affinity ligands for at least one binding site; degradation advantage, especially for multifunctional proteins with catalytic and scaffolding roles (e.g., IRAK4 [270], SHP2 [271]); and established therapeutic precedent, including targets such as AR [272], ER [273], and BTK [274]. These criteria may determine whether a target can successfully move from bench to clinic.

Among these targets, kinases remain one of the most intensively studied classes, supported by rich inhibitor libraries and well‐characterized disease associations. For example, BTK reached Phase III trials (e.g., BGB‐16673) [275], demonstrating high efficacy in B‐cell malignancies. EGFR and FGFR degraders further show how mutation selectivity can be incorporated into degrader design, thereby overcoming on‐target resistance while limiting toxicity [276, 277]. The challenge, however, is less about generating new degraders than about establishing a clear clinical rationale and differentiation from the extensive repertoire of existing kinase inhibitors. Hormone receptors, by contrast, represent the most clinically advanced area. AR and ERα degraders such as ARV‐110, CC‐94676, and ARV‐471 have already progressed to pivotal trials [272, 278, 279], offering advantages over traditional antagonists by eliminating variants such as AR‐V7 [280] and ligand‐binding domain mutants that underlie resistance. Linker design and E3 ligase selection remain central for optimizing breadth and potency. Equally critical will be improvements in oral bioavailability and tissue distribution, which determine whether applications can expand beyond oncology, as illustrated by dermatology programs like GT20029 [281]. These developments highlight how receptor‐targeting strategies can serve as a model for more rapid clinical translation when therapeutic precedent is well established.

Beyond these relatively mature categories, PROTACs are beginning to tackle nuclear proteins long regarded as “undruggable,” including Myc, β‐catenin, and STAT3 [282, 283, 284]. By removing such drivers directly, degraders can disrupt transcriptional programs in ways that inhibitors cannot achieve. Yet the hurdles are substantial: nuclear delivery remains difficult, and ligands must be highly selective to avoid widespread perturbation of gene expression. Advances in structural biology and computational discovery have improved prospects, though progress here continues to lag behind kinases and receptors. Antiapoptotic and mutant oncoproteins represent another promising area, with Bcl‐xL degraders limiting platelet toxicity by selectively targeting tumor cells [285], and KRAS^G12C and G12D degraders addressing both catalytic and scaffolding functions [286, 287, 288]. These cases illustrate the potential of targeted degradation to circumvent long‐standing liabilities of inhibitors, including toxicity and acquired resistance. At the same time, the difficulty of identifying ligands for smooth or dynamic protein surfaces remains one of the most persistent obstacles.

Epigenetic regulators such as BRD4, EZH2, and CBP/p300 are also compelling targets, given their central role in transcriptional reprogramming [289, 290, 291]. Degraders in this space offer opportunities to bypass compensatory pathways that limit inhibitors, although their broad activity increases the risk of off‐target epigenomic effects. Expanding beyond epigenetic proteins, immune‐related proteins are also under exploration. In immuno‐oncology, PD‐L1, PD‐1 (via peptide‐based PROTACs), and STAT3 degraders may help overcome checkpoint resistance [292], though systemic delivery of peptide agents remains particularly challenging. This domain arguably represents one of the most dynamic but also uncertain frontiers of PROTAC research—scientifically exciting, yet clinically demanding. Moving forward, progress will depend heavily on advances in computational triaging, the diversification of available E3 ligases, and the development of innovative delivery systems such as nanoparticle‐ or antibody‐conjugated PROTACs. These factors will be decisive in broadening the accessible target space and ensuring that preclinical discoveries are converted into durable therapeutic benefit.

## 5 PROTACs in the Clinic: Current Landscape and Leading Programs

An increasing number of PROTAC programs are entering clinical trials, demonstrating strong momentum in the field. According to US FDA clinical trial registry data, over 50 PROTAC candidates are currently under evaluation across various clinical phases (Table 2). These investigational programs span multiple important therapeutic targets, including AR, ER, BTK, and IRAK4 [293]. The clinical stage distribution follows a typical drug development pattern: most programs remain in early‐phase trials (Phase I and Phase I/II), reflecting the relatively novel nature of this technology. Simultaneously, several leading programs have successfully advanced to mid‐ and late‐stage clinical trials (Phase II and III), marking PROTAC technology's steady progress toward clinical applications [181].

**TABLE 2:** Clinical landscape of PROTAC therapeutics.

PROTAC	NCT number	Phase	Status	Target	Objectives	Preliminary findings	References
ARV‐471	NCT05930925	I	Completed	ERα	Healthy volunteers	Not reported	
	NCT05538312	I	Completed	ERα	Healthy volunteers	Not reported	[294]
	NCT04072952	I/II	Active, not recruiting	ERα	Breast cancer	Not reported	[295]
	NCT06125522	I/II	Active, not recruiting	ERα	Breast cancer	Not reported	
	NCT05501769	I	Active, not recruiting	ERα	Breast cancer	Not reported	[296]
	NCT05573555	I/II	Active, not recruiting	ERα	Breast cancer	Not reported	
	NCT05732428	I	Completed	ERα	Breast cancer	Not reported	[297]
	NCT05549505	II	Completed	ERα	Breast cancer	Not reported	[298]
	NCT05548127	I/II	Active, not recruiting	ERα	Breast cancer	Not reported	[299]
	NCT05673889	I	Completed	ERα	Healthy volunteers	Not reported	[300]
	NCT05652660	I	Completed	ERα	Healthy volunteers	Not reported	[300]
	NCT05909397	III	Active, not recruiting	ERα	Breast cancer	Significant PFS benefit in ESR1‐mutated patients; well‐tolerated	[301]
	NCT05463952	I	Active, not recruiting	ERα	Breast cancer	Not reported	[301]
	NCT05654623	III	Active, not recruiting	ERα	Breast cancer	Enhanced PFS in the ESR1‐mutated subgroup	[302]
	NCT06347861	I	Completed	ERα	Healthy volunteers	Not reported	
	NCT06911788	I	Completed	ERα	Healthy volunteers	Not reported	
	NCT06645938	I	Completed	ERα	Healthy volunteers	Not reported	
	NCT06005688	I	Completed	ERα	Healthy volunteers	Not reported	
	NCT06256510	I	Completed	ERα	Healthy volunteers	Not reported	[303]
	NCT06206837	I/II	Active, not recruiting	ERα	Breast cancer	Not reported	[304]
	NCT06275841	I	Completed	ERα	Healthy volunteers	Not reported	
ARV‐110	NCT03888612	II	Completed	AR	Prostate cancer	PSA50 response 16% overall, 46% in AR T878A/H875Y mutants	[305]
	NCT05177042	I	Completed	AR	Prostate cancer	Not reported	[306]
ARV‐766	NCT05067140	I/II	Active, not recruiting	AR	Prostate cancer	Pan‐AR degradation; 51% PSA50 in AR LBD mutants	[307]
CC‐94676	NCT06764485	III	Recruiting	AR	Prostate cancer	Ongoing rechARge study; rPFS primary endpoint	[308]
	NCT04428788	I	Active, not recruiting	AR	Prostate cancer	Preliminary efficacy and manageable safety in heavily	[309]
	NCT06417229	I	Completed	AR	Healthy volunteers	Not reported	
	NCT06433505	I	Completed	AR	Healthy volunteers	Not reported	
AC176	NCT05241613	I	Terminated	AR	mCRPC	Not reported	
	NCT05673109	I	Terminated	AR	mCRPC	Not reported	
HP518	NCT06155084	I/II	Recruiting	AR	mCRPC	Not reported	
	NCT05252364	I	Completed	AR	mCRPC	most AEs Grade 1–2	[2]
HRS‐5041	NCT05942001	I	Recruiting	AR	Prostate cancer	Not reported	
	NCT06559007	I	Completed	AR	Prostate cancer	Not reported	
	NCT07014488	I	Not yet recruiting	AR	Prostate cancer	Not reported	
	NCT06568094	I/II	Recruiting	AR	Prostate cancer	Not reported	
	NCT06738745	II	Not yet recruiting	AR	Prostate cancer	Not reported	
	NCT07115446	I	Not yet recruiting	AR	Prostate cancer	Not reported	
	NCT06830850	I	Recruiting	AR	mCRPC	Not reported	
GT20029	NCT06692465	II	Completed	AR	Androgenetic alopecia and acne vulgaris	Met primary endpoint; good safety and efficacy	[310]
	NCT06468579	I	Completed	AR	Acne, alopecia	Not reported	
	NCT05428449	I	Completed	AR	Androgenetic alopecia, acne vulgaris	Good safety/tolerability	[311]
AH‐001	NCT06927960	I	Recruiting	AR	Androgenetic alopecia	Not reported	
KT‐474	NCT06058156	II	Active, not recruiting	IRAK4	Atopic dermatitis	≥95% IRAK4 degradation in blood and skin	[312]
	NCT04772885	I	Completed	IRAK4	Healthy volunteer, atopic dermatitis, hidradenitis suppurativa	Good safety/tolerability	[313]
	NCT06028230	II	Active, not recruiting	IRAK4	Hidradenitis suppurativa	Not reported	[314]
KT‐413	NCT05233033	I	Completed	IRAK4, IKZF1/3	Non‐Hodgkin lymphoma, DLBCL	Good safety/tolerability	[315]
KT‐333	NCT05225584	I	Completed	STAT3	Liquid and solid tumors, T cell lymphomas	31.4% ORR in 51 patients	[316]
KT‐621	NCT06945458	I	Recruiting	STAT6	Atopic dermatitis	Rapid STAT6 degradation in blood cells	[317]
	NCT06673667	I	Recruiting	STAT6	Healthy volunteers	Not reported	
KT‐253	NCT05775406	I	Completed	MDM2	High grade myeloid malignancies, acute lymphocytic, leukemia, lymphoma, solid tumors	Tolerability	[318]
NX‐2127	NCT04830137	I	Recruiting	BTK, IKZF1/3	Relapsed/Refractory B‐cell malignancies	80% BTK degradation at 100 mg dose; preliminary antitumor activity	[319]
NX‐5948	NCT06717269	I	Recruiting	BTK	Healthy volunteers	Not reported	
	NCT06691828	I	Completed	BTK	Healthy volunteers	Not reported	
	NCT06593457	I	Completed	BTK	Healthy volunteers	Not reported	
	NCT05131022	I	Recruiting	BTK	Relapsed/refractory B‐cell malignancies	84.2% ORR in 49 patients	[320]
HSK29116	NCT04861779	I	Unknown status	BTK	Relapsed/refractory B‐cell malignancies	Not reported	[321]
BGB‐16673	NCT05294731	I/II	Recruiting	BTK	B‐cell malignancy, non‐Hodgkin lymphoma, mantle cell lymphoma	Not reported	
	NCT07005713	I	Recruiting	BTK	Chronic spontaneous urticaria	Not reported	
	NCT06634589	I/II	Recruiting	BTK	B‐cell malignancy, relapsed cancer, refractory cancer	Not reported	[322]
	NCT06973187	III	Active, not recruiting	BTK	CLL, SLL	78% ORR in CLL, 94% at 200 mg dose	[323]
	NCT05006716	I/II	Recruiting	BTK	B‐cell malignancy, marginal zone lymphoma, follicular lymphoma	Not reported	[324]
	NCT06970743	III	Recruiting	BTK	CLL, SLL	Not reported	
	NCT06846671	III	Recruiting	BTK	CLL	Not reported	
DT‐2216	NCT04886622	I	Recruiting	BCL‐X	T cell lymphomas	Not reported	[325]
	NCT06620302	I/II	Completed	BCL‐X	Childhood fibrolamellar carcinoma, recurrent childhood fibrolamellar carcinoma, recurrent childhood malignant solid neoplasm	Not reported	[326]
	NCT06964009	I	Recruiting	BCL‐X	Ovarian cancer	Not reported	
CFT‐8634	NCT05355753	I	Terminated	BRD9	Synovial sarcoma, soft tissue sarcoma	Failed to show efficacy in heavily pretreated patients	[327]
FHD‐609	NCT04965753	I	Terminated	BRD9	Synovial sarcoma	Grade 4 QTc prolongation led to clinical hold	[328]
CFT‐1946	NCT05668585	I	Active, not recruiting	BRAF V600	Solid tumors, melanoma, NSCLC	Avoids paradoxical RAF activation	[329]
CG001419	NCT06636500	I	Recruiting	NTRK	Healthy volunteer, pain management	Not reported	
PRT3789	NCT06682806	II	Recruiting	SMARCA2	Advanced solid tumor, esophageal cancer, metastatic solid tumor	Tumor shrinkage in NSCLC and esophageal cancer	[330]
	NCT05639751	I	On hold	SMARCA2	Advanced solid tumor, NSCLC	Tumor shrinkage in NSCLC and esophageal cancer	[331]
PRT7732	NCT06560645	I	Recruiting	SMARCA2	Advanced solid tumor, NSCLC	Not reported	[332]
MT‐4561	NCT06943521	I/II	Recruiting	BRD4	Advanced solid tumors	Not reported	
LT‐002‐158	NCT06932003	I/II	Not yet recruiting	IRAK4	Hidradenitis suppurativa	Not reported	
	NCT06082323	I	Recruiting	IRAK4	Healthy volunteer, hidradenitis suppurativa, atopic dermatitis	Not reported	
	NCT06931990	I/II	Not yet recruiting	IRAK4	Atopic dermatitis	Not reported	
TQB3019	NCT06943677	I	Recruiting	BTK	Advanced malignant cancer	Active against both BTK WT and C481S; exhibits excellent metabolic stability and favorable oral bioavailability	[333]
AUTX‐703	NCT06846606	I	Recruiting	KAT2A/KAT2B	Myelodysplastic syndromes, acute myeloid leukemia	Not reported	
PT0253	NCT06797336	I	Recruiting	Kras G12D	Solid tumor	Not reported	
BG‐60366	NCT06685718	I	Recruiting	EGFR	NSCLC	Not reported	
CFT8919	NCT06641609	I	Not Yet Recruiting	EGFR	NSCLC	Not reported	[334]
BTX‐9341	NCT06515470	I	Recruiting	CDK4/6	Breast cancer	Not reported	
ARV‐393	NCT06393738	I	Recruiting	BCL6	Non‐Hodgkin lymphoma, angioimmunoblastic T‐cell lymphoma	Not reported	[335]
ASP4396	NCT06364696	I	Recruiting	Kras G12D	Solid tumor	Not reported	[336]
ASP3082	NCT05382559	I	Recruiting	Kras G12D	Solid tumor	Selective degradation of KRAS G12D in preclinical models	[337]
BGB‐45035	NCT06342713	I	Recruiting	IRAK4	Healthy volunteers	Not reported	
	NCT07100938	II	Recruiting	IRAK4	Rheumatoid arthritis	Not reported	
BMS‐986458	NCT06090539	I/II	Recruiting	BCL6	Relapsed/refractory non‐Hodgkin lymphoma	Increased CD20 expression; tumor regression in preclinical models	[338]
AXT‐1003	NCT05965505	I	Terminated	EZH2	Relapsed/refractory non‐Hodgkin lymphoma	Early termination; reasons undisclosed	[339]
	NCT06484985	I	Recruiting	EZH2	Non‐Hodgkin lymphoma, advanced solid tumor	Not reported	
HSK40118	NCT06050980	I	Recruiting	EGFR	NSCLC	Not reported	[340]
AC676	NCT05780034	I	Recruiting	BTK	Relapsed/refractory B‐cell malignancies	Not reported	[341]
AC682	NCT05489679	I	Terminated	ERα	Breast cancer	Not reported	[342]
	NCT05080842	I	Terminated	ERα	Breast cancer	Not reported	[342]

*Abbreviations*: PFS, progression‐free survival; ESR1, estrogen receptor 1; PSA50, prostate‐specific antigen 50% decline; AR, androgen receptor; LBD, ligand‐binding domain; rPFS, radiographic progression‐free survival; mCRPC, metastatic castration‐resistant prostate cancer; AEs, adverse events; IRAK4, interleukin‐1 receptor‐associated kinase 4; STAT6, signal transducer and activator of transcription 6; ORR, overall response rate; BTK, Bruton's tyrosine kinase; CLL, chronic lymphocytic leukemia; SLL, small lymphocytic lymphoma; NSCLC, non‐small cell lung cancer; KRAS, Kirsten rat sarcoma viral oncogene homolog; DLBCL, diffuse large B cell lymphoma; ERα, estrogen receptor α.

The first PROTAC molecule, ARV‐110, entered clinical trials in 2019—a watershed moment that marked PROTAC technology's transition from the laboratory bench to actual clinical practice [343]. The success of ARV‐110 set off a wave of activity between 2020 and 2022, with numerous pharmaceutical companies jumping into the field and launching their own PROTAC programs [344]. This created a period of rapid pipeline growth. Then, in 2023 and 2024, we started seeing the leading programs move into pivotal trials, really setting the stage for commercial viability.

By mid‐2025, three PROTAC drugs have made it to Phase III trials with US FDA: vepdegestrant (ARV‐471), catadegbrutinib (BGB‐16673, NCT06973187), and gridegalutamide (BMS‐986365, CC‐94676—going after ER, BTK, and AR, respectively [345]. What is really exciting is that vepdegestrant has already submitted its marketing application to US FDA based on Phase III data in patients with previously treated ER+/HER2− 346], ESR1‐mutated advanced or metastatic breast cancer, potentially becoming the world's first approved PROTAC drug. This development timeline is remarkable: from the first PROTAC entering clinical trials in 2019 to the first drug submission in 2025 represents just 6 years, fully demonstrating PROTAC technology's tremendous potential and the pharmaceutical industry's strong endorsement of this revolutionary approach.

Overall, PROTAC technology has evolved from early proof‐of‐concept stages to a mature technology platform with clear commercialization prospects. The breakthrough of three drugs entering Phase III trials and the first NDA submission marks PROTAC technology's imminent entry into the industrialization era.

### 5.1 Hormone Receptor Degraders—The Most Mature Clinical Domain: AR Degraders

AR degraders represent a significant breakthrough in PROTAC technology for treating genitourinary malignancies. Prostate cancer, one of the most common male malignancies [347], is closely associated with abnormal AR signaling pathway activation. While existing AR antagonists like enzalutamide and abiraterone demonstrate excellent clinical performance, most patients receiving androgen deprivation therapy ultimately develop castration‐resistant prostate cancer (CRPC) [348]. This resistance often relates to AR overexpression, mutations, or splice variant generation. PROTAC degraders offer a new strategy by directly eliminating AR protein (Table 2).

#### 5.1.1 ARV‐110

As the world's first AR–PROTAC entering clinical trials, ARV‐110 employs classic bifunctional molecular design, with one end binding AR and the other recruiting E3 ubiquitin ligase CRBN to achieve AR‐specific degradation through ternary complex formation. In Phase I/II clinical trials (NCT03888612), ARV‐110 demonstrated favorable safety characteristics. At the recommended Phase II dose (420 mg once daily), major adverse reactions included nausea (42%), fatigue (27%), and vomiting (23%), with no Grade 4 or higher treatment‐related adverse events observed [349, 350]. Regarding efficacy, data through August 2021 [272] showed an overall PSA50 response rate of 16% and PSA30 response rate of 29% among 140 evaluable patients. Notably, 26 patients carrying AR T878A/S and/or H875Y mutations showed significantly higher response rates: PSA50 response rate reached 46% and PSA30 response rate reached 58%, while 114 patients without these mutations had corresponding response rates of only 10 and 23%. This finding provides important evidence for biomarker‐based precision therapy strategies.

#### 5.1.2 CC‐94676 (BMS‐986365)

CC‐94676 (also known as BMS‐986365) is the second PROTAC molecule globally to enter Phase III. In Phase I first‐in‐human clinical trials (CC‐94676‐PCA‐001, NCT04428788), BMS‐986365 demonstrated preliminary efficacy and manageable safety in heavily pretreated metastatic CRPC (mCRPC) patients [351]. The trial did not reach maximum tolerated dose, with the most common treatment‐related adverse reactions being asymptomatic QTc prolongation and bradycardia, primarily occurring in the first two cycles. Clinical and biochemical responses were observed in patients with or without AR gene LBD mutations, supporting evaluation in all patients regardless of AR receptor mutation status. Posthoc analysis showed patients unexposed to prior chemotherapy and not using opioid treatment had longer radiographic progression‐free survival (rPFS), supporting further development in mCRPC patients unexposed to chemotherapy with asymptomatic or mildly symptomatic disease. The ongoing rechARge study (NCT06764485) is a global Phase III, prospective, multicenter, randomized, adaptive, two‐part, open‐label trial planning to enroll approximately 960 mCRPC patients, evaluating BMS‐986365 versus investigator's choice of treatment (docetaxel or alternative ARPI like abiraterone or enzalutamide) [351]. The primary endpoint is rPFS, with trial initiation in March 2025.

#### 5.1.3 ARV‐766

ARV‐766 is Arvinas’ second‐generation AR degrader, a novel, potent oral PROTAC AR degrader. Compared with first‐generation ARV‐110, ARV‐766 incorporates three key structural optimizations: replacing the middle six‐membered ring with a substituted four‐membered ring, substituting the chlorine atom on the left aromatic ring with methoxy, and replacing fluorinated thalidomide with fluorinated benzamide, which serves as a CRBN ligand with high selectivity and plasma stability. ARV‐766's important breakthrough lies in its pan‐AR degradation capability, degrading AR LBD mutations including AR L702H, H875Y, and T878A, while ARV‐110 cannot degrade AR L702H mutations, which account for approximately 11% of metastatic CRPC patients [352] and represent a major resistance mechanism. In the ongoing Phase I/II clinical trial (NCT05067140), as of April 15, 2024, 123 patients received ARV‐766 treatment, with 53 carrying AR LBD mutations. In Phase I dose escalation studies, ARV‐766 showed no dose‐limiting toxicity at 20–500 mg once daily, with no maximum tolerated dose reached; 100 and 300 mg were selected as Phase II recommended doses. Among 47 evaluable PSA AR LBD mutation patients, 51% achieved PSA decline ≥30% and 43% achieved PSA decline ≥50%. Among 20 evaluable soft tissue lesion AR LBD mutation patients, objective response rate was 30%. Major treatment‐related adverse reactions included fatigue (33%), nausea (20%), and diarrhea (15%), mostly mild to moderate, with only nine patients (7%) requiring dose reduction and 10 patients (8%) discontinuing due to adverse reactions [353]. Based on these excellent clinical data, particularly significant efficacy in patients carrying AR LBD mutations, ARV‐766 is considered likely to enter Phase III clinical trials ahead of ARV‐110.

#### 5.1.4 Other AR–PROTAC Programs in Development

Beyond the major programs above, multiple AR–PROTAC candidates are currently in development, including Accutar Biotech's AC‐0176 (NCT05241613) [354], Hinova Pharmaceuticals’ HP518 (NCT05252364) [2], and HRS‐5041 (NCT06559007) entering Phase I, which, while still in early development stages, collectively constitute diversified technological positioning in the AR–PROTAC field.

#### 5.1.5 Dermatology Indications—Innovative Exploration of Expanded Applications

AR–PROTAC applications in dermatology represent an important expansion of TPD technology into nononcology indications. Kintor Pharmaceutical's GT20029, as the world's first topical PROTAC compound entering clinical trials [355], innovatively targets both androgenetic alopecia and acne simultaneously, based on the common pathophysiological mechanism of excessive AR activation in hair follicles and sebaceous glands. GT20029 employs topical administration to maximize drug concentration at lesion sites while significantly reducing systemic exposure, thereby minimizing potential systemic adverse reactions. In completed Phase I clinical trials, GT20029 demonstrated good safety and tolerability in both China and the United States, with major adverse reactions limited to mild local irritation. In 2024, GT20029's multicenter, randomized, double‐blind, placebo‐controlled Phase II clinical trial (NCT06692465) successfully met its primary endpoint [310]. This trial randomized 180 male androgenetic alopecia patients for 12‐week treatment, showing GT20029 achieved statistically significant and clinically meaningful efficacy, marking an important breakthrough for PROTAC technology in dermatological applications. Besides GT20029, other AR–PROTAC projects based on similar principles are in development, such as AH‐001 (NCT06927960) [356], collectively constituting diversified positioning for PROTAC technology applications in dermatology and providing strong evidence for this innovative technology platform's clinical translation into broader therapeutic areas.

### 5.2 ERα Degraders—Breast Cancer Breakthrough ARV‐471 (NCT05654623, Phase III)—The PROTAC Drug Closest to Market

ERα degraders represent the most promising breakthrough in PROTAC technology for solid tumor treatment, with this field forming a complete pipeline from proof‐of‐concept to late‐stage clinical development (Table 2). According to latest clinical trial registry analysis, currently two PROTAC drugs specifically targeting ERα have entered clinical development, with ARV‐471 (vepdegestrant) having entered pivotal Phase III clinical trials, becoming the PROTAC drug closest to market [181], while AC682 as an emerging candidate is showing potential in Phase I clinical trials (NCT05489679) [357]. ARV‐471, as the world's first PROTAC molecule entering Phase III clinical trials, holds milestone significance in breast cancer treatment. This drug achieves selective ERα degradation through CRBN E3 ligase, with its unique mechanism potentially overcoming traditional endocrine therapy resistance. In the VERITAC‐2 pivotal Phase III clinical trial (NCT05654623), the study enrolled 624 patients with ER+/HER2− advanced breast cancer previously treated with CDK4/6 inhibitors and endocrine therapy, with primary endpoint of PFS [358]. Trial results showed that in the ESR1‐mutated patient subgroup, vepdegestrant significantly prolonged PFS compared with fulvestrant, with median PFS of 5.0 months (95% CI: 3.7–7.4) versus 2.1 months (95% CI: 1.9–3.5), hazard ratio 0.58 (95% CI: 0.43–0.78, *p* < 0.001), meeting the prespecified primary endpoint. However, in the overall intent‐to‐treat population, median PFS was 3.8 versus 3.6 months, respectively, hazard ratio 0.83 (95% CI: 0.69–1.01, *p* = 0.07), not reaching statistical significance. These results indicate vepdegestrant provides clear clinical benefit in ESR1‐mutated patients, providing important evidence for precision medicine. From a safety perspective, vepdegestrant demonstrated relatively favorable tolerability. In the safety analysis population, 86.9% of patients experienced adverse events of any grade, 23.4% experienced Grade 3 or higher adverse events, with only 2.9% discontinuing treatment due to adverse events. Most common adverse events included fatigue (26.6%), AST elevation (14.4%), ALT elevation (14.4%), and nausea (13.5%), mostly Grade 1–2 mild to moderate events [359]. Notably, QT prolongation occurred in 9.9% of patients, but no serious arrhythmia complications were observed, providing important safety data for clinical application.

### 5.3 BTK Degraders

#### 5.3.1 BGB‐16673 (NCT06973187, Phase III)

BGB‐16673, as the most promising BTK–PROTAC, is the first such drug entering Phase III clinical trials, marking an important milestone for BTK degrader technology [360]. In early clinical trials, BGB‐16673 demonstrated encouraging efficacy data. Among 49 treated relapsed/refractory CLL patients, overall response rate (ORR) reached 78%, with the 200 mg dose group achieving 94% ORR. More importantly, BGB‐16673 showed broad antitumor activity in other hematologic malignancies: 90% ORR in Waldenström's macroglobulinemia patients (21 patients), 50% ORR in follicular lymphoma (eight patients), and 67% ORR in marginal zone lymphoma (eight patients) [323]. These results highlight BGB‐16673's broad activity potential as a first‐in‐class BTK degrader in B‐cell malignancies.

#### 5.3.2 NX‐2127 (NCT04830137, Phase I)

NX‐2127 [361] is a multitarget PROTAC simultaneously targeting BTK and IKZF1/3 proteins, embodying PROTAC technology's unique advantage of simultaneously degrading multiple pathogenic proteins. This drug is currently in Phase I clinical trials evaluating safety and efficacy in B‐cell malignancies.

#### 5.3.3 NX‐5948 (NCT06717269, Phase I)

NX‐5948 [362] specifically targets BTK protein degradation, with indications expanded to B‐cell malignancies and autoimmune diseases, reflecting BTK PROTAC application potential across different disease areas.

#### 5.3.4 Other Candidates

HSK29116 (NCT04861779), TQB3019 (NCT06943677), and AC676 (NCT05780034) are all in Phase I clinical trials targeting different types of B‐cell malignancies, forming a complete BTK PROTAC drug clinical development pipeline [321, 363, 364].

### 5.4 IRAK4 Degraders

IRAK4 degraders represent an important breakthrough in immune inflammatory disease treatment, with significant mechanistic advantages over traditional small molecule inhibitors. IRAK4, as a key component of the MyD88 complex, not only possesses kinase activity but also serves important scaffolding functions, while traditional IRAK4 kinase inhibitors only block kinase activity without completely inhibiting scaffolding function. Protein degraders achieve more complete target inhibition by utilizing cellular UPSs to completely eliminate IRAK4 protein, simultaneously eliminating both kinase and scaffolding functions. KT‐413 is a bifunctional molecule that not only degrades IRAK4 but also simultaneously degrades IMiD substrates Ikaros and Aiolos, currently in Phase I clinical trials for MYD88‐mutated tumors (NCT05233033) [365, 366]. KT‐474 (SAR444656) is the first selective IRAK4 degrader entering autoimmune indication clinical trials, focusing on immune inflammatory skin diseases, currently in Phase II clinical trials (NCT06058156) [367]. In Phase I clinical trials, KT‐474 achieved ≥93% blood IRAK4 degradation with single doses of 600—1600 mg in healthy volunteers, and ≥95% IRAK4 degradation with continuous 14‐day daily dosing of 50—200 mg. In HS and AD patients, KT‐474 not only achieved similar blood IRAK4 degradation effects but also normalized overexpressed IRAK4 levels in skin lesions and significantly improved disease‐related inflammatory biomarkers [313]. Other drugs are also in development, with LT‐002‐158 in Phase I/II clinical trials (NCT06932003) for indications including acne, autoimmune diseases, hidradenitis suppurativa, and atopic dermatitis. BGB‐45035 is in Phase I clinical trials for atopic dermatitis and prurigo nodularis (NCT06342713). These programs’ successful advancement validates IRAK4 degradation therapeutic potential in hematologic malignancies and autoimmune diseases, providing important proof‐of‐concept for TPD technology applications across different disease areas.

### 5.5 KRAS G12D Degraders

KRAS occupies a special position in cancer research history. For over 40 years since its discovery by Harvey and Kirsten in the 1960s [368, 369], KRAS has been considered an “undruggable” target. This is primarily because KRAS protein has a relatively smooth spherical structure, lacking deep hydrophobic pockets and obvious binding sites, with GTP binding affinity reaching picomolar levels while intracellular GTP concentrations are as high as 0.5 µM, making competitive inhibitor development nearly impossible [370]. A major breakthrough occurred in 2013 when researchers discovered a compound capable of binding a small pocket in KRAS G12C mutant protein, reigniting efforts to develop active inhibitors [371]. In May 2021, US FDA approved the first KRAS G12C inhibitor sotorasib (Lumakras), marking the historic transition of KRAS from “undruggable” to “druggable” [372, 373]. However, KRAS G12D, as one of the most common KRAS mutation subtypes (accounting for approximately 34% of pancreatic ductal adenocarcinoma patients, 12% of colorectal cancer patients, and 4% of lung adenocarcinoma patients), still faces enormous challenges in targeted therapy due to lacking cysteine residues similar to G12C for covalent binding [374]. Protein degradation technology provides new possibilities for overcoming this bottleneck, with three major KRAS G12D degrader programs currently advancing clinical development:

ASP3082 is the first KRAS G12D proteasome‐targeting chimeric degrader entering clinical trials [375], selectively targeting KRAS G12D mutant protein for degradation by recruiting E3 ubiquitin ligase proteins. In preclinical studies, ASP3082 selectively degraded KRAS G12D mutant protein and showed growth inhibitory activity in KRAS G12D mutant cancer cells while having no effect on KRAS wild‐type cancer cells. When administered weekly intravenously to mice transplanted with KRAS G12D mutant cancer cells, it demonstrated significant antitumor effects. Currently in Phase I clinical trials for solid tumors including pancreatic, colorectal, and non‐small cell lung cancers (NCT05382559), using 3–12 patient dose escalation cohorts with 21‐day cycle intravenous administration and tumor‐specific expansion cohorts enrolling ≤20 participants per tumor type [376].

ASP4396 from the same company is another KRAS G12D degrader designed to target KRAS G12D mutant protein degradation through the UPS, potentially providing higher efficacy and safety than inhibitors by blocking both enzymatic activity and scaffolding functions with higher target selectivity [336]. Currently in Phase I clinical trials for locally advanced or metastatic solid tumors (NCT06364696), using open‐label, multicenter, dose escalation and dose expansion design, enrolling adult patients with ≥1 measurable lesion and ECOG performance status 0 or 1.

PT0253 is an emerging program that, according to preclinical comparative data, shows best‐in‐class potential and may overcome limitations of current clinical‐stage KRAS inhibitors, currently in Phase I clinical trials for KRAS G12D mutant advanced solid tumors (NCT06797336) [377]. These KRAS G12D degrader programs represent important attempts to conquer “undruggable” targets, potentially providing more effective and durable treatment options for KRAS‐driven tumors with extremely poor prognosis like pancreatic cancer through complete elimination of mutant proteins rather than simple functional inhibition.

### 5.6 EGFR Degraders

EGFR degraders represent an innovative therapeutic strategy for overcoming traditional EGFR inhibitor resistance, showing tremendous clinical value in NSCLC treatment. Traditional EGFR inhibitors like gefitinib, erlotinib, and osimertinib have achieved significant efficacy in EGFR‐mutated NSCLC patients but inevitably develop acquired resistance, mainly caused by secondary mutations like T790M and C797S [378]. EGFR degraders potentially overcome this therapeutic bottleneck by completely eliminating mutant EGFR proteins through TPD rather than simply blocking kinase activity.

CFT8919 is a representative program in this field—a potent and selective EGFR L858R degrader based on allosteric EGFR binding motifs, developed using the company's proprietary BiDAC degradation platform technology [379]. In preclinical studies, CFT8919 as monotherapy showed activity in EGFR L858R‐driven NSCLC models both in vitro and in vivo, and importantly remained effective in models carrying secondary resistance mutations (like EGFR T790M and C797S) [380], suggesting CFT8919 may be effective in patients with resistance due to EGFR secondary mutations. Additionally, CFT8919 demonstrated intracranial activity, suggesting potential for treating brain metastases, which is particularly important for NSCLC patients since brain metastases are common and poor‐prognosis complications. This drug is currently in Phase I clinical trials for NSCLC (NCT06641609) [381]. Beyond CFT8919, other drugs like BG‐60366 (NCT06685718) and HSK40118 (NCT06050980) are also in Phase I clinical trials evaluating safety and efficacy in NSCLC. The emergence of EGFR degraders not only provides new therapeutic approaches for solving EGFR inhibitor resistance but, more importantly, their potential as monotherapy in first‐line settings may avoid secondary EGFR mutations commonly seen with currently approved EGFR inhibitors, potentially providing more durable treatment benefits for EGFR‐mutated NSCLC patients.

### 5.7 BCL6 Degraders

BMS‐986458 is a first‐in‐class highly selective BCL6 ligand‐directed degrader currently in Phase I/II clinical trials (NCT06090539) treating follicular lymphoma and diffuse large B‐cell lymphoma. Preclinical data show this drug demonstrates antitumor effects in 80% of BCL6‐expressing NHL cell lines in vitro, increasing CD20 expression up to 20‐fold within 72 h, which is significant for relapsed/refractory disease [382]. In vivo studies showed daily oral administration achieved tumor regression, and when combined with anti‐CD20 drugs, achieved tumor‐free status in 70% of animal models. The 28‐day canine toxicity study showed good tolerability. Its multimodal mechanism not only directly acts on tumor cells but also modulates follicular helper T cells in the immune microenvironment, potentially becoming a first‐in‐class chemotherapy‐free treatment option for B‐cell NHL patients. ARV‐393 is in earlier Phase I clinical trials (NCT06393738) for treating B‐cell non‐Hodgkin lymphoma and angioimmunoblastic T‐cell lymphoma, currently still evaluating safety and preliminary efficacy [383].

### 5.8 BRD4 and BRD9 Targeting: Innovative Strategies and Clinical Challenges

MT‐4561 is a novel BRD4 degrader currently undergoing Phase II clinical evaluation. (NCT06943521) for BRD4‐dependent solid tumors, covering broad tumor types including NSCLC, breast cancer, and prostate cancer.

BRD9 is a key protein in chromatin regulatory systems, participating in BAF complex functional regulation, playing crucial roles in cancers like synovial sarcoma and SMARCB1‐deficient tumors [384]. However, since traditional bromodomain inhibitors cannot effectively treat BRD9‐dependent cancers, this target has long been considered an “undruggable” difficult target. CFT‐8634 and FHD‐609 are both innovative PROTAC/BiDAC protein degraders that exert antitumor effects by degrading rather than inhibiting BRD9 [327, 328]. CFT‐8634 received US FDA orphan drug designation, while FHD‐609 is an intravenous BRD9 degrader, both showing good activity against BRD9‐dependent tumors in preclinical studies.

However, while CFT‐8634 was well‐tolerated in clinical trials (NCT05355753) and achieved high‐level BRD9 degradation, it failed to produce sufficient single‐agent efficacy in heavily pretreated patients, leading to formal development termination in November 2023 and complete trial closure in Q1 2024 [327]. FHD‐609 experienced a Grade 4 QTc prolongation serious cardiac toxicity event in a synovial sarcoma patient at the second‐highest dose in its Phase I clinical trial (NCT04965753), leading US FDA to implement partial clinical hold in April 2023 [385]. Subsequently, no plans to independently advance dose expansion studies were made, effectively terminating further development. These two cases fully demonstrate the significant challenges faced in balancing safety and efficacy in TPD drug development.

### 5.9 SMARCA2 Targeting Twin Stars: PRT3789 and PRT7732

PRT3789 [386] is the first highly selective intravenous SMARCA2 degrader entering clinical trials, while PRT7732 is a potent oral SMARCA2 degrader. Both drugs’ development is based on the same scientific principle: SMARCA4 is frequently mutated in multiple cancers, including up to 10% of NSCLC [387, 388]. SMARCA4‐deficient cancer cells are highly dependent on SMARCA2 for survival, and targeting SMARCA2 in SMARCA4‐deficient cancers is expected to produce strong synthetic lethal effects [232].

In a Phase I (NCT05639751) study investigating the novel SMARCA2 degrader PRT3789, patients with advanced cancers harboring SMARCA4 deficiency were treated to evaluate safety and efficacy [389]. The trial population included individuals with various tumor types, with NSCLC being the predominant diagnosis, followed by pancreatic and breast malignancies. Treatment‐emergent adverse events of Grade 3 or higher occurred in about half of the patients; however, only a minority of these events were linked to the drug itself. Common side effects reported included nausea, appetite loss, and fatigue. Among those patients whose responses could be evaluated, encouraging tumor shrinkage was primarily seen in NSCLC and esophageal cancer cases, while patients with other tumor types did not exhibit measurable tumor regression.

### 5.10 STAT3 and STAT6 Targeting

KT‐333 is a STAT3‐targeting PROTAC degrader using VHL‐dependent mechanisms, currently in Phase Ia/1b clinical trials (NCT05225584) for indications including relapsed/refractory B‐cell and T‐cell lymphomas, classical Hodgkin lymphoma, solid tumors, and large granular lymphocytic leukemia/T‐cell prolymphocytic leukemia, showing 31.4% ORR among 51 enrolled patients [390].

KT‐621 is the world's first STAT6‐targeting PROTAC drug, showing good pharmacokinetic characteristics in healthy subjects with rapid degradation of STAT6 protein in blood immune cells [391]. Doses of 12.5 mg once daily and above achieved rapid degradation effects. While STAT6 protein degradation in skin tissue required longer time to reach >90%, the drug showed good safety, with ideal metabolic and activity data and oral administration advantages. Currently in Phase I clinical trials (NCT06945458) evaluating atopic dermatitis patients, awaiting further clinical data validation of efficacy in patient populations.

### 5.11 PROTAC Drugs Targeting Other Protein Targets Under Clinical Evaluation

As PROTAC technology continues developing, new PROTAC drugs keep emerging, providing new strategies for cancer treatment through protein degradation mechanisms. DT‐2216 [392], as a BCL‐X‐targeting protein degrader, is currently in Phase I clinical trials in T‐cell lymphoma patients evaluating safety and preliminary efficacy (NCT04886622). EZH2‐targeting drug AXT‐1003 [339, 393] was originally designed for treating relapsed or refractory non‐Hodgkin lymphoma, but its Phase I clinical trial was terminated early (NCT05965505), with specific termination reasons undisclosed.

In hematologic malignancy treatment, AUTX‐703 [394] targets KAT2A and KAT2B, currently in Phase I studies in myelodysplastic syndromes and acute myeloid leukemia patients (NCT06846606), while KT‐253 [395], as an MDM2 protein degrader, restores p53 tumor suppressor function by inducing selective MDM2 protein degradation, currently in Phase I clinical evaluation in multiple blood cancers (NCT05775406).

In solid tumor treatment, CFT‐1946 represents an important technological breakthrough as a novel bifunctional degradation activating compound (BIDAC) that simultaneously inhibits and degrades mutant BRAF V600 protein. Compared with traditional BRAF inhibitors, CFT‐1946's unique advantage lies in avoiding paradoxical RAF activation phenomena, since degraded BRAF V600 mutant protein cannot form dimeric signaling complexes. This drug is selective for mutant protein without affecting wild‐type BRAF, currently in Phase I/II clinical studies for multiple BRAF V600‐mutated solid tumors including melanoma [396], NSCLC, colorectal cancer, and anaplastic thyroid cancer, with both monotherapy and combination therapy with MEK inhibitor trametinib (NCT05668585).

Additionally, BTX‐9341 [397] targets CDK4/6, currently in Phase I trials in breast cancer patients (NCT06515470), while CG001419, as an NTRK‐targeting drug specifically for treating advanced solid tumors carrying NTRK alterations, is in Phase I/II clinical studies (NCT06636500).

### 5.12 Clinical Challenges and Future Development Directions for PROTAC Drugs

Despite remarkable progress in recent years, with vepdegestrant and other candidates entering Phase III trials and even submitting NDAs, this revolutionary technology still faces severe challenges on the path to commercialization [181]. PROTAC drugs consist of heterobifunctional molecules comprising target protein ligands, linkers, and E3 ubiquitin ligase ligands. While peptide PROTACs exist in research, all PROTACs in current clinical trials are composed of small molecules, yet their molecular weights far exceed traditional “small molecule” drug concepts.

Oral bioavailability represents the largest technical bottleneck for PROTAC drugs. Since PROTAC molecular weights typically exceed 800 Da, far beyond ideal ranges for oral drugs, they universally possess unfavorable oral absorption characteristics including high molecular weight, multiple hydrogen bond donors/acceptors, and high polar surface area. For example, ARV‐110's recommended Phase II dose is 420 mg once daily, with only 23.8% oral bioavailability in rats [398]. This low bioavailability not only requires higher doses, increasing patient burden, but also raises “hook effect” concerns, where excessive concentrations form nonproductive binary complexes rather than ternary complexes, actually reducing target protein degradation efficiency.

Safety challenges are equally concerning, with recent terminations of multiple PROTAC programs due to serious safety issues serving as warnings for the entire field. FHD‐609 [328], a BRD9 degrader, experienced Grade 4 QTc prolongation and syncope in Phase I trials, ultimately leading to US FDA clinical hold. CFT‐8634 [327], despite achieving high‐level BRD9 degradation, failed to produce sufficient efficacy in heavily pretreated patients and was discontinued. These cases suggest PROTAC drugs may have issues including off‐target protein degradation, inadvertent degradation of essential cardiomyocyte proteins, and complex balancing of efficacy versus safety.

Facing these challenges, future PROTAC technology development requires multidimensional innovative breakthroughs. At the molecular design level, researchers are working on developing shorter, more rigid linkers to reduce molecular flexibility, exploring novel E3 ligase ligands for smaller, more stable alternatives, and introducing prodrug strategies to improve bioavailability through in vivo activation. Delivery technology innovation is also becoming important, including using lipid nanoparticles (LNPs) for enhanced stability, developing ADCs for precise targeting, and local administration strategies following GT20029's [355] successful dermatological application.

For safety assessment, establishing more comprehensive cardiac toxicity evaluation models, developing PROTAC‐specific safety assessment indicators, utilizing humanized organ‐on‐chip technology for toxicity prediction, and constructing AI‐based toxicity prediction models are current research focuses. Next‐generation PROTAC technology development is also anticipated, including reversible PROTAC molecular design, multitarget PROTAC strategies, and novel E3 ligase development beyond traditional VHL, CRBN, and IAP systems [399, 400, 401]. Long‐term, PROTAC technology success depends not only on individual drug approvals but on establishing a sustainable technology platform. As a completely new drug mechanism, PROTACs face unprecedented regulatory challenges, requiring new assessment standards covering target protein degradation quantitative evaluation, long‐term safety monitoring, and special population safety assessment. Commercially, high development costs and relatively high failure rates require pharmaceutical companies to develop precise market positioning, reasonable pricing strategies, and comprehensive pharmacoeconomic data within limited patent protection periods.

Despite current multiple challenges, with advancing science and technology and accumulating clinical experience, issues including oral bioavailability, safety, and mechanistic understanding are expected to be gradually resolved.

## 6 Delivery Systems for PROTACs: Challenges and Strategic Solutions

**FIGURE 3:**
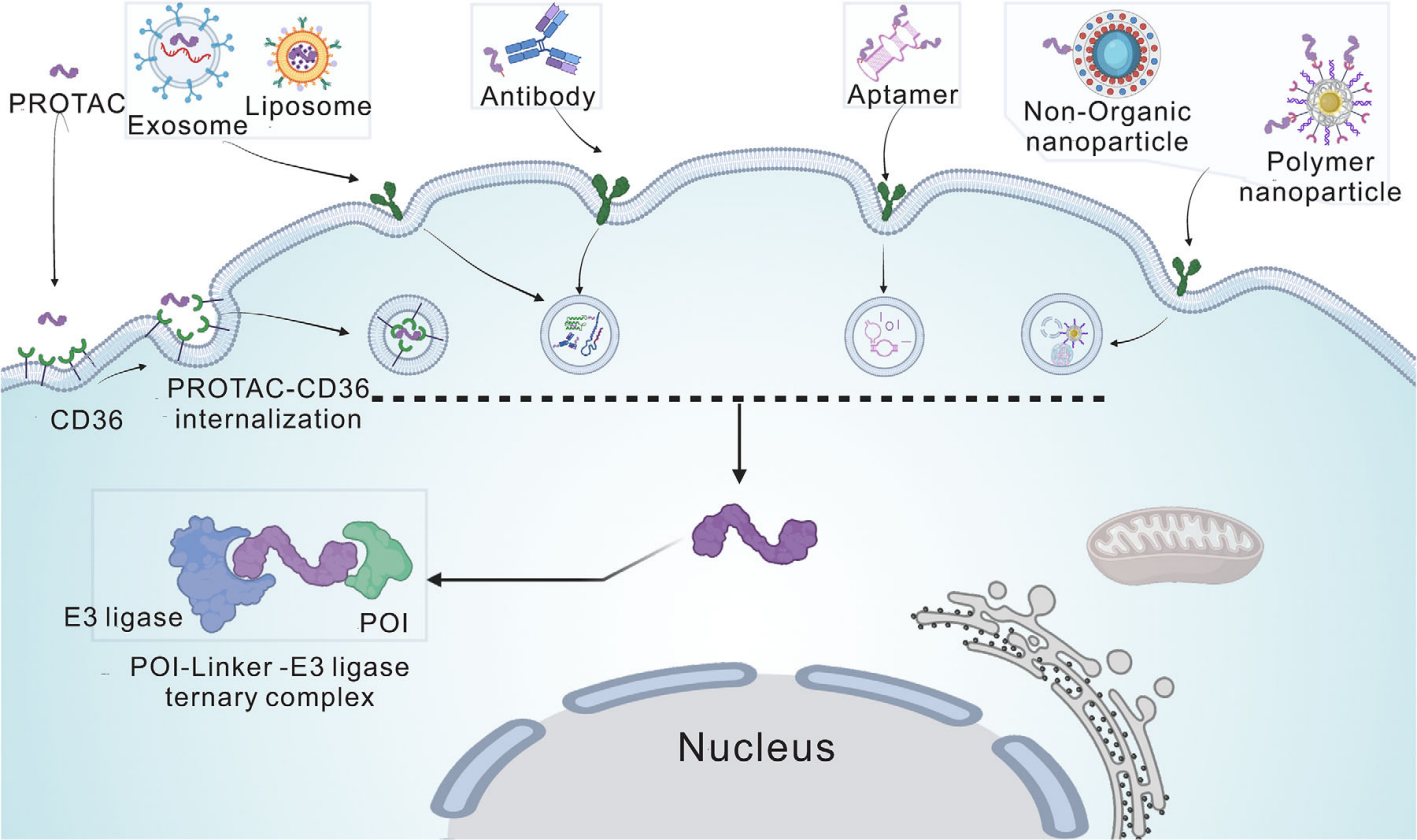
Advanced delivery strategies for PROTACs. Delivery platforms, including exosomes, liposomes, antibodies, aptamers, and polymeric/nonorganic nanoparticles, have been developed to enhance the stability, bioavailability, tissue specificity, and cellular uptake of PROTACs.

PROTACs, as an emerging class of targeted protein degraders, have demonstrated immense therapeutic potential surpassing traditional inhibitors in a series of clinical trials. However, effectively delivering PROTACs to intracellular targets and ensuring their stability and bioavailability in complex biological environments remain core challenges that must be overcome to fully unleash their pharmacological activity. PROTAC molecules are often larger and more complex than traditional small molecule drugs, which leads to difficulties in crossing biological membranes, evading biological barriers, and avoiding nonspecific binding. To overcome these delivery hurdles, researchers have developed a variety of innovative strategies, which can be broadly categorized into two main approaches: structural optimization of the PROTAC molecule itself to enhance its cell permeability, and the use of various advanced delivery systems for its encapsulation or conjugation (Figure 3).

### 6.1 Optimizing PROTAC Molecules for Enhanced Cell Permeability

The passive diffusion of traditional small molecule drugs into cells generally adheres to the Ro5, where parameters such as molecular weight, lipophilicity, and the number of hydrogen bond donors and acceptors should fall within specific ranges to ensure good oral bioavailability and cell permeability. However, many PROTAC molecules, particularly chimeras linking two or more functional domains, often have molecular weights that exceed the Ro5 limits, falling into the eRo5 or even bRo5 categories [402]. Such molecules typically exhibit lower passive membrane permeability, leading to insufficient intracellular concentrations and, consequently, limiting their in vivo efficacy.

To address this issue, a “chemical endocytic medicinal chemistry” strategy has been proposed, aiming to actively promote PROTAC entry into cells via endocytic pathways through structural modifications [402]. This approach no longer solely relies on passive diffusion but instead leverages the cell's intrinsic membrane transport mechanisms. For instance, a key study identified CD36, a fatty acid transporter, as a membrane receptor for PROTACs, especially eRo5/bRo5 molecules, mediating their cellular uptake [402]. By optimizing the structure of PROTACs to enhance their binding affinity to CD36, their intracellular internalization efficiency and pharmacological activity can be significantly improved, without compromising their solubility or stability. This strategy of finely tuning PROTAC structures to adapt to cellular endocytosis mechanisms offers a promising avenue for overcoming the permeability bottleneck of large PROTAC molecules. By identifying and exploiting specific receptor‐mediated endocytosis, more efficient and targeted intracellular delivery of PROTACs can be achieved, which is crucial for improving their bioavailability and targeting.

### 6.2 Advanced Delivery Systems: Constructing Multifunctional Vehicles

In addition to structural optimization of the PROTAC molecule itself, encapsulating or conjugating PROTACs into various advanced delivery systems is another major category of strategies for addressing their delivery challenges. These systems aim to protect PROTACs from enzymatic degradation, improve their solubility, prolong their systemic circulation time, and achieve targeted delivery to specific cells or tissues (Figure 3).

#### 6.2.1 Conjugate‐Based Delivery Systems

Conjugate delivery strategies achieve precise targeting and efficient delivery by covalently linking PROTAC molecules to biomolecules with specific delivery functions.

##### 6.2.1.1 Antibody–PROTAC Conjugates

Drawing on the successful experience of ADCs, DACs leverage the high specificity of antibodies to recognize specific cell surface antigens. Antibodies can precisely bind to receptors on the surface of target cells and mediate the internalization of the conjugated PROTACs into cells via receptor‐mediated endocytosis. The advantage of this strategy lies in its excellent cell and tissue specificity, which can effectively reduce the accumulation of PROTACs in nontarget cells, thereby lowering systemic toxicity, while enhancing PROTAC enrichment at target sites to improve efficacy. DACs represent a powerful platform for achieving precise tumor‐targeted delivery of PROTACs. Maneiro et al. [3] demonstrated the successful development of trastuzumab–PROTAC conjugates that selectively degrade BRD4 only in HER2‐positive breast cancer cells while sparing HER2‐negative cells, achieving stable drug‐to‐antibody ratios of 4 with excellent serum stability. Similarly, Wang et al. [403] developed ROR1‐targeting DACs that showed enhanced tumor‐specific BRD4 degradation and improved antitumor efficacy in PC3 and MDA–MB‐231 xenograft models, with superior pharmacokinetic profiles including extended half‐life compared with unconjugated PROTACs. The conjugation strategy employed click chemistry and site‐specific cysteine engineering to ensure controlled PROTAC release while maintaining antibody binding specificity. Notably, combination therapy with immune checkpoint inhibitors demonstrated enhanced therapeutic outcomes through tumor microenvironment modulation and increased T cell infiltration [403]. These studies establish DACs as promising candidates for overcoming traditional limitations of PROTAC selectivity and pharmacokinetics in cancer therapy.

##### 6.2.1.2 Aptamer–PROTAC Conjugates

This emerging conjugation strategy utilizes nucleic acid molecules, such as aptamers or engineered oligonucleotides, as PROTAC delivery vehicles. Aptamers are nucleic acid molecules obtained through in vitro selection that can bind to specific target molecules with high affinity and specificity [404]. By conjugating PROTACs to aptamers that target cell surface receptors, receptor‐mediated endocytosis of PROTACs can be achieved, thereby specifically delivering them to target cells. This strategy combines the high specificity recognition capabilities of nucleic acids with the degradation function of PROTACs, offering new possibilities for precision medicine. Recent advances have demonstrated the feasibility of APCs for tumor‐selective protein degradation. Zhang et al. [405] developed ZL216, a novel PROTAC using nucleic acid aptamer AS1411 as a targeting ligand for nucleolin, which showed excellent serum stability and water solubility while achieving potent nucleolin degradation in breast cancer cells both in vitro and in vivo. Similarly, Sun et al. [406] reported conjugated polyelectrolyte/ssDNA hybrid polyplexes that combined cationic conjugated polyelectrolytes with aptamer sequences, demonstrating enhanced cellular uptake efficiency and nuclease resistance for targeted ERα degradation. These studies demonstrate that nucleic acid aptamers can effectively construct PROTACs, broadening the PROTAC toolbox and providing promising strategies for developing tumor‐selective protein degraders with improved delivery efficiency and reduced off‐target effects.

#### 6.2.2 Nanoparticle‐Based Delivery Systems

Nanoparticles, as versatile drug delivery platforms, have garnered significant attention due to their nanoscale size, high drug loading capacity, and modifiability. They can effectively encapsulate PROTACs, protecting them from enzymatic degradation and rapid clearance, while promoting cellular uptake and targeted delivery through various mechanisms.

##### 6.2.2.1 Liposomes

Liposomes, versatile vesicles composed of one or more lipid bilayers surrounding an aqueous core, are highly effective carriers for delivering PROTACs due to their ability to encapsulate both hydrophilic and hydrophobic molecules. For PROTACs, which often suffer from poor water solubility, low bioavailability, and limited cell permeability due to their large molecular weight (>800 Da) and polar surfaces, liposomes offer a promising solution by encapsulating these molecules within the lipid bilayer or aqueous compartment. This encapsulation enhances in vivo stability, protects PROTACs from premature degradation, and minimizes nonspecific interactions with biological systems, thereby reducing off‐target effects and toxicity [404]. Recent studies have demonstrated significant improvements in PROTAC pharmacokinetic properties through liposomal delivery. For GNE‐01 and GNE‐02, PROTAC‐in‐cyclodextrin liposome formulations showed 80‐fold and 23‐fold enhancements in AUC, respectively, compared with solution formulations [407]. Additionally, innovative codelivery approaches have been developed, such as DTX/ARV‐lip systems that coencapsulate BRD4–PROTAC ARV825 with docetaxel, demonstrating enhanced antitumor effects through dual mechanisms of protein degradation and chemotherapy with tumor inhibition rates reaching 57.4% [408]. The biocompatibility, high loading capacity, and tunable physicochemical properties of lipid‐based nanoparticles make them ideal for improving the pharmacokinetic profiles of PROTACs, facilitating their clinical translation. A notable example of liposome‐based PROTAC delivery is the development of LARPC, a nanoliposome coloading the protein kinase C inhibitor palmitoyl‐dl‐carnitine chloride (PC) and the BRD4‐targeting PROTAC ARV‐825 [409]. Furthermore, engineered bioPROTAC formats capable of complexing with cationic and ionizable lipids via electrostatic interactions have shown remarkable efficacy, with near‐complete elimination (up to 95% clearance) of targets within hours of treatment when delivered by biocompatible LNPs, demonstrating successful degradation of proteins localized to various subcellular compartments including the mitochondria, nucleus, cytosol, and membrane [75]. In this system, the hydrophobic ARV‐825 resides within the lipid bilayer alongside the palmitoyl chain of PC, forming nanoparticles with a size of approximately 105.25 ± 2.76 nm and a positive surface charge of 26.6 mV. These properties enable selective tumor accumulation via the EPR effect and enhance internalization into cancer cells, resulting in potent inhibition of melanoma cell lines. This demonstrates the potential of liposomes to maximize anticancer efficacy while minimizing side effects through targeted delivery. Liposomes can be engineered to further optimize PROTAC delivery. Surface modifications, such as PEGylation, extend circulation time by reducing clearance, while conjugation with targeting ligands—such as antibodies, peptides, or small molecules—enables active targeting of specific cell types, such as cancer cells overexpressing FOLR1 or HER2 [404].

##### 6.2.2.2 Polymer‐Based Nanostructures

These nanoparticles are fabricated from biodegradable polymers (e.g., PLGA, PLA), possessing good biocompatibility and controlled release characteristics. Polymeric NPs can encapsulate PROTACs in the form of micelles, nanospheres, or nanocapsules, providing drug protection and sustained release effects. Their surfaces are easily functionalized, allowing for attachment of targeting ligands to achieve specific cellular uptake, or to trigger PROTAC release in response to the tumor microenvironment. Recent advances demonstrate that stereocomplexation of P(LLA‐stat‐EtGly) and P(DLA‐stat‐EtGly) can tune melting temperature and crystallinity of polymers, directly impacting mechanical properties and drug release behavior without affecting hydrophilic/hydrophobic balance [410]. The formation of stereocomplexes leads to stronger polymer chain interactions, increased thermal stability, and higher Young's modulus compared with homochiral crystals [410]. MZ1‐loaded polymeric nanoparticles using US FDA‐approved PLA and PEI demonstrated excellent properties with particle sizes of 114–124 nm, encapsulation efficiency of 55.7%, and high stability over 7 days [411]. These nanoparticles exhibited biphasic release patterns with controlled diffusion, while stereocomplexation kinetics showed that 7‐day formation time optimized nanoparticle crystallinity and mechanical properties [410, 411]. Trastuzumab‐conjugated MZ1 nanoparticles (MZ1–ACNPs) showed significantly enhanced cytotoxic effects in HER2+ breast cancer cells and overcame natural MZ1 resistance in resistant cell lines, while antibody conjugation prevented protein corona formation and maintained delivery specificity [411].

##### 6.2.2.3 Exosome‐Based Nanoparticles

Exosome‐mediated PROTAC delivery systems represent a revolutionary drug delivery paradigm that demonstrates tremendous potential in the field of antiviral therapeutics. PROTACs constitute a class of bifunctional molecules capable of harnessing the cellular UPS for selective degradation of target proteins, while exosomes serve as naturally occurring nanoscale extracellular vesicles that possess excellent biocompatibility, stability, and the capacity to traverse biological barriers, thereby providing an optimal carrier platform for targeted PROTAC delivery [40, 412]. Exosome‐mediated PROTAC delivery significantly enhances drug bioavailability, enables cell‐specific targeting, and minimizes off‐target effects to the greatest extent possible. Through engineered modification of exosomal surface ligands, such as expression of cancer‐specific surface receptors like EGFR, precise delivery to specific cell populations can be achieved, including HIV‐infected cells and viral reservoirs [413]. Methods for loading PROTACs into exosomes encompass coincubation, electroporation, and sonication techniques, with electroporation demonstrating superior loading efficiency while preserving exosomal structural integrity [414]. This delivery system is particularly well‐suited for targeting the degradation of critical HIV proteins including Tat, p24, and viral proteases, thereby disrupting viral replication and assembly processes. Exosomes possess the remarkable ability to penetrate immune‐privileged sites, enabling access to and clearance of latent HIV reservoirs that are typically refractory to existing therapeutic modalities. As next‐generation PROTAC technologies continue to advance, exosome‐mediated delivery systems hold considerable promise for playing increasingly important roles in the treatment of HIV and other viral diseases.

##### 6.2.2.4 Nonorganic Nanoparticles

Inorganic NPs, such as gold NPs, silica NPs, and magnetic NPs, have also been explored for PROTAC delivery. These inorganic nanomaterials generally possess good biocompatibility, ease of surface modification, and controllable physicochemical properties. By modifying the surface of NPs with amino or thiol groups, PROTACs can be conjugated and enter cells via endocytosis. A notable example is the development of NIR‐activatable PROTAC nanocages using UCNPs‐based MSNs. In this system, a photocaged PROTAC targeting BRD4 was loaded into UMSNs to construct NIR light‐activatable nanocages that could be activated upon 980 nm laser irradiation to release active PROTAC for TPD and cancer cell apoptosis [415]. This approach addresses the limitations of UV–visible light‐controlled PROTACs by utilizing NIR light, which offers better tissue penetration and reduced phototoxicity. The nanocages demonstrated effective BRD4 degradation and successfully suppressed tumor growth with 58.7% tumor growth inhibition in vivo. Another innovative approach involves injectable thermosensitive nanocomposite hydrogels that integrate multiple therapeutic modalities. Wu et al. [416] developed a system comprising CCM‐coated MSNs containing both a peptide‐based BMI1 PROTAC and paclitaxel, combined with imiquimod‐encapsulated CaCO3 NPs within a PLGA–PEG–PLGA polymer framework. This system exploits the immunosuppressive TME by simultaneously targeting BMI1 degradation while modulating immune cell populations through pH‐responsive release of immunoadjuvants. Upon injection, the hydrogel undergoes in situ gelation and gradually degrades in the acidic TME, releasing therapeutic NPs in a sustained manner. The CaCO3 NPs decompose under acidic conditions, neutralizing tumor pH and releasing imiquimod to promote M2‐to‐M1 macrophage polarization and DC maturation, thereby enhancing antitumor immune responses. This approach demonstrated superior therapeutic efficacy in HNSCC models, effectively suppressing both primary tumor growth and metastasis. Inorganic NPs may also offer additional functionalities, such as imaging or photothermal therapy, enabling integrated diagnosis and treatment.

PROTAC delivery research is advancing toward multifunctional, intelligent, and targeted approaches. From the structural optimization of PROTAC molecules themselves to a diverse array of advanced delivery systems, each strategy offers a unique solution to the specific challenges faced in PROTAC delivery. Future research will likely combine the advantages of multiple strategies to develop more efficient and safer PROTAC delivery platforms, ultimately propelling these revolutionary drugs toward clinical application.

#### 6.2.3 Key Challenges and Future Perspectives: Critical Insights on PROTAC Delivery Systems

After thoroughly examining the current landscape of PROTAC technology, it is become clear that the field has reached a pivotal juncture. The ability to solve delivery challenges will ultimately determine whether these promising molecules can achieve their transformative potential. In our view, the delivery system puzzle represents both the most formidable barrier and the most exciting opportunity for innovation in the PROTAC arena. The fundamental challenge arises from an inherent paradox: PROTACs need to be large enough to simultaneously engage both the target protein and E3 ligase, yet must retain sufficient drug‐like properties for effective delivery. When molecular weights routinely exceed 1000 Da, conventional small molecule delivery principles simply fall short [417]. We have seen numerous elegantly designed PROTACs that demonstrate exceptional potency in vitro fail to progress clinically due to poor bioavailability and inadequate tissue penetration. This disconnect between laboratory promise and clinical reality demands a paradigm shift in how we approach PROTAC delivery.

Among the various delivery strategies explored, each presents distinct advantages and limitations that require careful consideration. Nanoparticle‐based systems, particularly LNPs, offer excellent protection against enzymatic degradation and enable targeted delivery through surface modifications [418]. However, these systems often suffer from rapid clearance in vivo, and their size constrains tumor penetration. Manufacturing complexity and batch‐to‐batch variability also pose significant hurdles for clinical translation and regulatory approval. AOCs represent an elegant approach for achieving cell‐specific delivery, leveraging the exquisite selectivity of antibodies. Yet in practice, conjugating an already complex PROTAC molecule to a 150 kDa antibody creates an unwieldy construct. The resulting conjugates face formulation challenges, potential immunogenicity concerns, and remain limited to targets accessible from the extracellular space. Moreover, the heterogeneous nature of these conjugates complicates quality control and pharmacokinetic optimization. Prodrug strategies offer another compelling approach, enabling PROTACs to traverse biological barriers in an inactive form before activation at the target site [419]. We find enzyme‐cleavable and pH‐responsive prodrugs that exploit the tumor microenvironment particularly intriguing [420]. Nevertheless, the additional molecular complexity introduced by prodrug moieties can push already large PROTACs beyond practical limits for oral administration. The kinetics of prodrug activation must also be carefully balanced to avoid premature activation or excessive stability.

Looking ahead, several key developments could revolutionize PROTAC delivery. First, integrating artificial intelligence and machine learning could enable prediction of optimal delivery strategies based on PROTAC structure and target tissue characteristics [243]. The potential for AI to identify novel cell surface receptors that mediate PROTAC uptake in specific tissues or disease states is particularly exciting. Second, combination delivery approaches will likely prove more successful than single‐strategy solutions. For instance, incorporating a prodrug–PROTAC into targeted nanoparticles could leverage multiple mechanisms for enhanced delivery. The key lies in maintaining the right balance between complexity and practicality. Third, tissue‐specific delivery will become increasingly important as PROTACs expand beyond oncology. Developing brain‐penetrant PROTACs for neurodegenerative diseases will require fundamentally different approaches than those used for solid tumors. Similarly, achieving selective delivery to inflamed tissues for autoimmune applications will demand innovative targeting strategies.

Solving the delivery challenge will require unprecedented collaboration between medicinal chemists, formulation scientists, clinicians, and regulatory experts. Based on the rapid progress we have witnessed and the creative solutions emerging from laboratories worldwide, we remain optimistic that while the delivery challenges facing PROTACs are formidable, they are ultimately surmountable.

## 7 Future Directions and Improvement Strategies for PROTAC Technology

**FIGURE 4:**
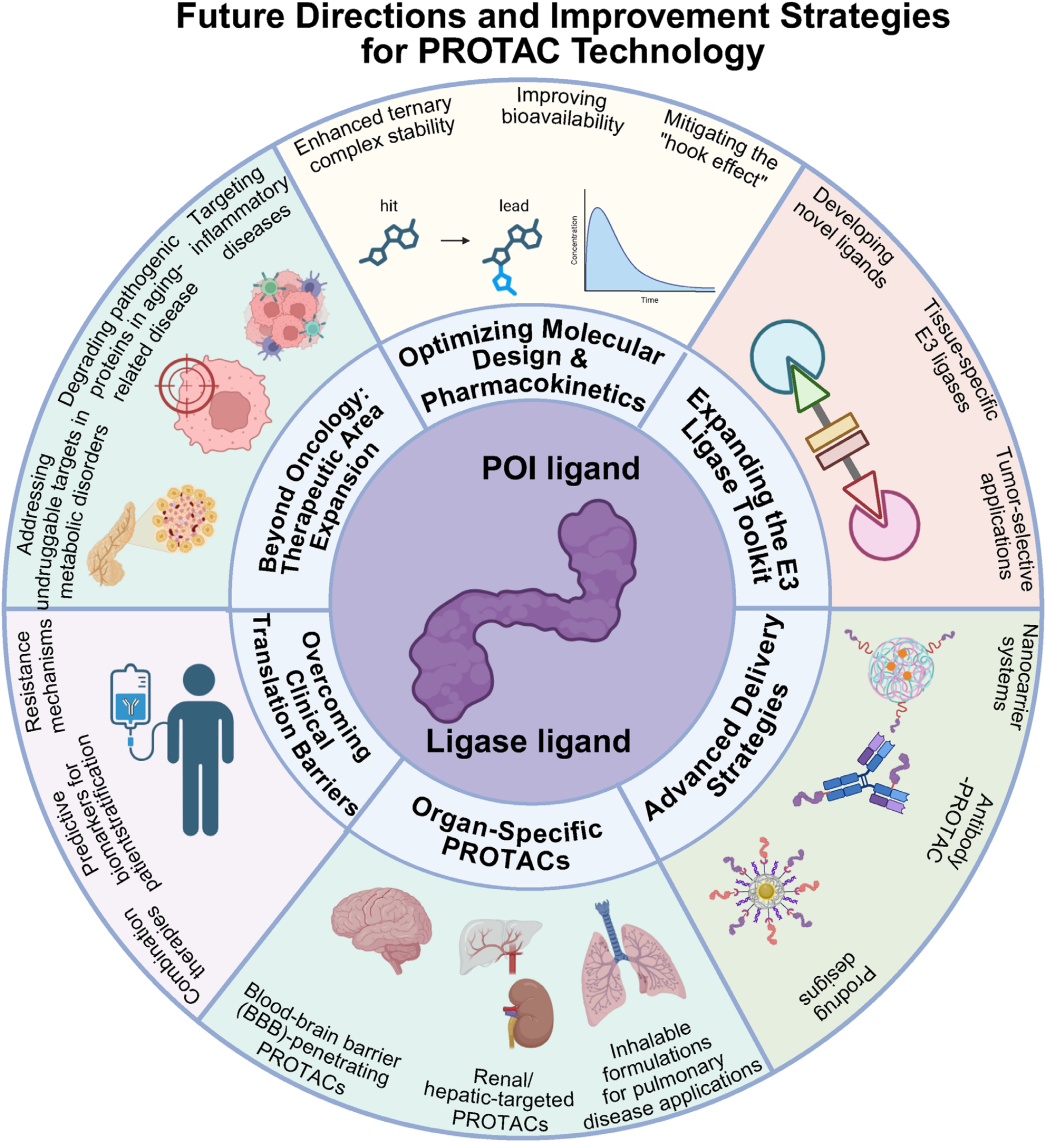
Future directions and improvement strategies for PROTAC technology. Emerging areas of development are highlighted, such as the expansion of the E3 ligase toolbox, organ‐specific targeting, advanced molecular designs, prodrug strategies, and combination therapies. These innovations aim to overcome current limitations and broaden the therapeutic applicability of PROTACs.

As PROTAC technology transitions from the proof‐of‐concept stage to the critical juncture of clinical application, the field is confronting unprecedented opportunities and challenges. While multiple PROTAC molecules have successfully entered clinical trials and demonstrated encouraging therapeutic efficacy, realizing their full potential as next‐generation precision medicine tools requires systematic optimization across multiple dimensions, including molecular design, delivery strategies, and clinical translation. Based on current technical bottlenecks and clinical needs, the future development of PROTAC technology can be categorized into six core directions. These developmental pathways will collectively propel TPD technology from laboratory settings toward broader clinical applications (Figure 4).

### 7.1 Molecular Design and Pharmacokinetic Optimization

#### 7.1.1 Strategies for Enhancing Ternary Complex Stability

The core mechanism of PROTAC molecules relies on forming stable ternary complexes (POI–PROTAC–E3 ligase), but most current PROTAC molecules form ternary complexes with limited stability, directly impacting protein degradation efficiency [421]. Future molecular design will focus on enhancing ternary complex stability through computational modeling and structural optimization. Emerging artificial intelligence‐assisted design methods, such as deep learning‐based protein–protein interaction prediction models, are providing powerful tools for rational PROTAC design. By accurately predicting spatial conformational relationships between POI, PROTAC, and E3 ligase, researchers can design linker structures with superior geometric compatibility, significantly improving ternary complex formation efficiency and stability.

#### 7.1.2 Improvement of Cellular Permeability and Oral Bioavailability

Traditional PROTAC molecules, due to their large molecular weight (typically exceeding 800 Da) and multiple hydrogen bond donors and acceptors, often face challenges of poor cell membrane permeability and low oral bioavailability [422]. This challenge is particularly pronounced in treating central nervous system diseases. To address this issue, researchers are exploring multiple innovative strategies. Among these, prodrug design represents one of the most promising approaches. By introducing modifying groups that can be cleaved by specific enzyme systems onto PROTAC molecules, this strategy not only improves drug ADME properties but also enables tissue‐specific drug activation [252]. Additionally, novel delivery technologies such as LNPs and cyclodextrin inclusion complexes provide new solutions for enhancing PROTAC molecule bioavailability.

#### 7.1.3 Mechanistic Elucidation and Circumvention of the “Hook Effect”

The “hook effect” represents a unique challenge for PROTAC technology, referring to the phenomenon where PROTAC molecules paradoxically exhibit decreased degradation efficiency at high concentrations [423]. The mechanism underlying this effect involves competitive formation of binary complexes (POI–PROTAC or PROTAC–E3), which cannot promote protein degradation and instead hinder effective ternary complex formation. Understanding the molecular mechanisms of the hook effect is crucial for PROTAC optimization design. Recent studies indicate that by precisely modulating the binding affinity ratio between POI ligands and E3 ligands, as well as optimizing linker length and flexibility, the effective concentration window of PROTAC molecules can be effectively expanded while reducing hook effect occurrence [424].

### 7.2 Expansion of E3 Ligase Toolbox

#### 7.2.1 Development of Novel E3 Ligase Ligands

Although the human genome encodes over 600 E3 ubiquitin ligases, current PROTAC design primarily relies on a few E3 ligases, such as CRBN, VHL, IAP, and MDM2. This limited E3 ligase selection significantly restricts the application scope and optimization space of PROTAC technology [425]. Future research priorities include discovering and developing small molecule ligands targeting novel E3 ligases. Through high‐throughput screening, fragment‐based drug discovery, and computer‐aided drug design, researchers are working to expand the E3 ligase toolbox available for PROTAC design. Development of novel E3 ligands not only provides new solutions for difficult‐to‐degrade target proteins but also enables more precise tissue‐specific protein degradation.

#### 7.2.2 Utilization of Tissue‐Specific E3 Ligases

Different tissues and cell types exhibit significant variations in E3 ligase expression patterns, providing a natural foundation for achieving tissue‐specific protein degradation. For example, certain E3 ligases are highly expressed in the nervous system while showing low expression in peripheral tissues [426]. PROTAC molecules designed using such E3 ligases hold promise for achieving brain‐specific protein degradation [427]. Similarly, aberrant expression patterns of certain E3 ligases in tumor cells provide opportunities for cancer‐specific therapy. Through systematic analysis of E3 ligase expression profile changes under different pathological conditions, researchers can identify disease‐specific E3 ligase targets, thereby designing PROTAC molecules with higher selectivity and lower toxicity.

#### 7.2.3 Implementation of Tumor‐Selective Applications

Tumor cells and normal cells exhibit systematic differences in E3 ligase expression, proteasome activity, cell cycle status, and other aspects [428]. These differences provide biological foundations for designing tumor‐selective PROTACs. For instance, overexpression of specific E3 ligases in certain tumor cells makes them more sensitive to corresponding PROTAC molecules, while normal cells remain relatively unaffected due to lower E3 ligase expression levels. Furthermore, unique characteristics of the tumor microenvironment (such as hypoxia, acidic pH, specific enzyme activities) can be utilized to design environment‐responsive PROTAC molecules, achieving selective protein degradation at tumor sites.

### 7.3 Development of Advanced Delivery Strategies

#### 7.3.1 Application of Nanocarrier Systems

Nanocarrier technology provides innovative solutions for addressing PROTAC molecule delivery challenges. Various nanodelivery systems, including LNPs, polymeric nanoparticles, and inorganic nanocarriers, are being utilized for PROTAC molecule encapsulation and delivery [429, 430]. These nanocarriers not only protect PROTAC molecules from enzymatic degradation in biological environments but also enable targeted delivery through surface modification. For example, by modifying nanocarrier surfaces with specific targeting ligands, PROTAC molecule enrichment in particular tissues or cell types can be achieved, thereby improving therapeutic efficacy while reducing systemic toxicity.

#### 7.3.2 Innovation in AOCs

AOCs represent an important developmental direction for TPD technology. By conjugating PROTAC molecules with specific antibodies, AOCs can achieve highly specific cellular targeted delivery [431]. When AOCs bind to cell surfaces expressing specific antigens, PROTAC molecules are delivered intracellularly through endocytosis, where they are released and exert protein degradation functions. This strategy is particularly suitable for treating cancer cells expressing specific surface markers, significantly improving treatment selectivity and efficacy.

#### 7.3.3 Refinement of Prodrug Design

Prodrug design holds unique value in PROTAC technology, not only improving drug physicochemical properties but also enabling conditional activation and targeted release [432]. Enzyme‐activated prodrugs utilize differences in specific enzyme activities between particular tissues or pathological states to achieve selective PROTAC molecule activation. Photo‐activated prodrugs enable spatiotemporally specific protein degradation through external light control. pH‐responsive prodrugs utilize acidic characteristics of tumor microenvironments to achieve tumor‐specific drug release. Development of these prodrug strategies provides powerful tools for precise PROTAC technology applications in complex biological systems.

### 7.4 Development of Organ‐Specific PROTACs

#### 7.4.1 Design of Blood–Brain Barrier Penetrating PROTACs

Treatment of central nervous system diseases has consistently been a major challenge for PROTAC technology, primarily due to strict blood–brain barrier (BBB) restrictions on macromolecular drugs [433]. To address this issue, researchers are exploring solutions from multiple angles. First is molecular design optimization, improving PROTAC molecule BBB penetration ability by reducing molecular weight, decreasing polar surface area, and optimizing hydrogen bond donor/acceptor ratios [434]. Second is utilizing carrier‐mediated transport systems, such as glucose transporters and amino acid transporters, achieving active transport by conjugating PROTAC molecules with substrates of these transporters. Additionally, nanodelivery systems and physical methods (such as focused ultrasound) provide new possibilities for PROTAC molecules to cross the BBB.

#### 7.4.2 Development of Kidney/Liver‐Targeted PROTACs

The kidneys and liver, as major metabolic and excretory organs, play crucial roles in the development and progression of numerous diseases. Developing organ‐specific PROTAC molecules holds significant importance for treating kidney diseases (such as renal fibrosis, glomerular diseases) and liver diseases (such as hepatic fibrosis, fatty liver disease) [435, 436]. Kidney targeting can utilize glomerular filtration characteristics and proximal tubule reabsorption mechanisms, achieving renal enrichment by modulating PROTAC molecule physicochemical properties such as molecular weight and charge distribution. Liver targeting can utilize specific receptors on hepatocyte surfaces (such as asialoglycoprotein receptors) or abundant hepatic blood flow characteristics.

#### 7.4.3 Application of Inhaled Formulations in Pulmonary Diseases

Treatment of pulmonary diseases such as pulmonary fibrosis, asthma, and lung cancer can achieve local high‐concentration drug exposure through inhalation administration while reducing systemic side effects [437]. PROTAC molecule inhaled formulation development requires consideration of drug aerosol properties, pulmonary deposition patterns, local clearance mechanisms, and other factors. By optimizing carrier systems (such as liposomes, microspheres) and delivery devices, effective PROTAC molecule delivery and sustained release in the lungs can be achieved. This administration route is particularly suitable for treating lung‐specific pathogenic proteins, such as fibrosis‐related proteins playing key roles in pulmonary fibrosis.

### 7.5 Overcoming Clinical Translation Barriers

#### 7.5.1 In‐Depth Study of Resistance Mechanisms

Compared with traditional small molecule inhibitors, PROTAC molecules face unique resistance challenges. PROTAC resistance may arise from multiple levels: target protein mutations leading to decreased PROTAC binding ability, E3 ligase expression or functional abnormalities, proteasome system dysfunction, insufficient intracellular PROTAC concentrations, and others [438, 439]. Understanding these resistance mechanisms is crucial for designing next‐generation PROTAC molecules and developing rational combination treatment strategies. By establishing resistant cell models and conducting genomic and proteomic analyses, researchers can identify key resistance drivers, providing molecular targets for overcoming resistance.

#### 7.5.2 Establishment of Predictive Biomarkers

Personalized PROTAC therapy requires establishment of effective predictive biomarker systems. These biomarkers should predict patient responsiveness to specific PROTAC treatments, including both efficacy and toxicity [440]. Candidate biomarkers include target protein expression levels, E3 ligase expression profiles, proteasome activity, genomic variations, and others. Through multiomics data integration analysis and machine learning methods, comprehensive predictive models can be constructed, providing scientific foundations for precision medicine applications of PROTAC therapy.

#### 7.5.3 Optimization of Combination Treatment Strategies

Combined application of PROTAC molecules with other therapeutic modalities holds tremendous potential. PROTACs can be combined with traditional chemotherapy drugs, targeted therapy drugs, immunotherapy drugs, radiotherapy, and others, improving therapeutic efficacy through synergistic actions of different mechanisms. For example, PROTAC degradation of antiapoptotic proteins can enhance chemotherapy sensitivity [441]; PROTAC degradation of immunosuppressive proteins can enhance immunotherapy efficacy [442]. Combination treatment strategy design requires consideration of drug interactions, administration timing, dose optimization, and other factors, necessitating validation through systematic preclinical studies and early clinical trials.

### 7.6 Expansion of Therapeutic Areas

#### 7.6.1 Targeted Treatment of Inflammatory Diseases

Inflammatory diseases such as rheumatoid arthritis, inflammatory bowel disease, and systemic lupus erythematosus involve complex inflammatory signaling networks, with many key regulatory proteins difficult to target with traditional drugs [443]. PROTAC technology provides new intervention approaches for these “undruggable” inflammatory regulatory proteins [159]. For example, transcription factor NF‐κB, STAT family proteins, inflammation‐related kinases, and others can serve as potential PROTAC targets. By degrading these key inflammatory regulatory proteins, PROTACs hold promise for achieving more precise and durable anti‐inflammatory effects.

#### 7.6.2 Protein Homeostasis Regulation in Age‐Related Diseases

The aging process is accompanied by functional decline of protein homeostasis systems, leading to accumulation of harmful proteins and loss of key functional proteins. PROTAC technology can exert antiaging effects by selectively degrading aging‐related harmful proteins (such as key proteins in senescent cell antiapoptotic pathways). For example, PROTAC degraders of antiapoptotic proteins BCL‐XL and BCL‐2 have demonstrated significant antiaging effects [444]. Additionally, PROTACs may be used to degrade aging‐related transcriptional regulators, metabolic enzymes, and others, providing new pharmacological intervention strategies for healthy aging and lifespan extension.

#### 7.6.3 Conquering Undruggable Targets in Metabolic Diseases

Metabolic diseases such as diabetes, obesity, and lipid metabolism disorders involve numerous metabolic regulatory proteins [445, 446], many of which lack suitable small molecule binding pockets and are considered “undruggable” targets. PROTAC technology provides entirely new intervention strategies for these targets through protein degradation mechanisms. For example, transcription factor SREBP, metabolic enzyme HMGCR, nuclear receptors, and others can be targeted for degradation through PROTAC technology [447, 448]. This approach not only effectively regulates metabolic pathways but may also avoid compensatory responses potentially generated by traditional inhibitors.

Future development of PROTAC technology will advance simultaneously along multiple dimensions, from fundamental molecular design optimization to complex clinical translation applications. Each developmental direction contains tremendous innovative potential. With continuous integration of cutting‐edge technologies such as artificial intelligence, nanotechnology, and precision medicine, PROTACs are poised to become core technological platforms for next‐generation precision therapy. However, realizing this vision requires close collaboration among academia, industry, and regulatory agencies to collectively propel PROTAC technology from laboratory to clinic, ultimately benefiting patients worldwide. The next decade will be a critical period for PROTAC technology development, and we have reason to believe that this revolutionary technology will bring unprecedented breakthroughs to human health endeavors.

## 8 Conclusion and Prospects

### 8.1 Transformative Impact and Current Achievements

The development of PROTACs represents a paradigmatic shift in drug discovery, fundamentally altering our approach to therapeutic intervention from traditional occupancy‐driven inhibition to event‐driven protein degradation. This comprehensive analysis reveals that PROTAC technology has successfully transitioned from academic curiosity to clinical reality within just two decades, achieving unprecedented milestones that validate its revolutionary potential. The successful progression of multiple PROTAC candidates through clinical trials, with vepdegestrant (ARV‐471) becoming the first to submit for US FDA approval based on Phase III data [181], demonstrates the technology's maturation and commercial viability. Across diverse therapeutic areas, from hormone‐dependent cancers and hematologic malignancies to inflammatory diseases and neurodegenerative conditions, PROTACs have consistently demonstrated their unique ability to target previously “undruggable” proteins, including transcription factors like STAT3 and MYC [449], mutant oncoproteins such as KRAS G12C and BRAF V600E, and scaffolding proteins that lack conventional binding pockets [450]. The clinical landscape analysis reveals remarkable progress, with over 50 PROTAC candidates currently under evaluation across various phases, spanning critical targets including AR, ER, BTK, and numerous kinases and epigenetic modulators [385]. The breakthrough discovery of CD36‐mediated [402] endocytic transport mechanisms has fundamentally resolved one of the field's most persistent challenges, cellular uptake of large, polar PROTAC molecules, opening new avenues for rational design and optimization. Furthermore, the development of innovative delivery strategies, including AOCs, tissue‐specific formulations, and prodrug approaches, has significantly expanded the therapeutic window and reduced systemic toxicity concerns that initially limited clinical translation.

### 8.2 Addressing Current Limitations and Emerging Solutions

Despite these remarkable achievements, PROTAC technology continues to face significant challenges that require systematic resolution for broader clinical adoption. The molecular weight and polarity constraints that limit oral bioavailability remain formidable obstacles, as evidenced by the relatively high doses required for many clinical candidates and the associated manufacturing complexities. The “hook effect” phenomenon, where excessive concentrations paradoxically reduce degradation efficiency, necessitates careful dose optimization and represents a unique pharmacological challenge not encountered with traditional therapeutics [451]. Additionally, the emergence of resistance mechanisms, while less common than with conventional inhibitors, poses long‐term sustainability concerns that demand proactive investigation and mitigation strategies. However, the field is actively addressing these limitations through multiple innovative approaches. The integration of artificial intelligence and machine learning in PROTAC design is revolutionizing our ability to predict ternary complex stability, optimize linker geometry, and enhance target selectivity. The expansion of the E3 ligase toolbox beyond traditional CRBN and VHL systems is providing new opportunities for tissue‐specific degradation and overcoming resistance mechanisms. Novel delivery technologies, including the chemical endocytic medicinal chemistry strategy based on CD36 transport, are systematically improving bioavailability while maintaining drug stability and potency. The development of reversible PROTACs, multitarget degraders, and conditional activation systems represents the next generation of sophisticated molecular tools that will further enhance therapeutic precision and safety profiles.

### 8.3 Future Directions and Transformative Potential

Looking ahead, PROTAC technology stands poised to expand into entirely new therapeutic territories that were previously considered intractable. The successful targeting of cellular senescence pathways through selective degradation of antiapoptotic proteins opens unprecedented opportunities for addressing age‐related diseases and extending healthspan. The development of brain‐penetrant PROTACs promises to revolutionize treatment of neurodegenerative diseases, where protein aggregation and misfolding represent central pathological mechanisms. In autoimmune and inflammatory diseases, the ability to selectively degrade key regulatory proteins like IRAK4 and STAT6 offers more precise intervention strategies compared with broad immunosuppression [452, 453]. The technological convergence of PROTAC platforms with emerging therapeutic modalities presents extraordinary synergistic potential. Combination strategies with immunotherapy, particularly through degradation of immunosuppressive proteins and immune checkpoint modulators, could fundamentally reshape cancer treatment paradigms. The integration of PROTAC technology with gene therapy and cell therapy approaches may enable unprecedented precision in therapeutic protein modulation. Furthermore, the development of organ‐specific and disease‐context‐selective PROTACs will likely enable personalized medicine approaches based on individual E3 ligase expression profiles and proteasome activity patterns.

### 8.4 Transforming Modern Medicine

The ultimate impact of PROTAC technology extends far beyond individual therapeutic applications, it represents a fundamental expansion of the druggable proteome and a new paradigm for addressing complex diseases. By enabling therapeutic intervention against proteins that have resisted conventional drug discovery efforts for decades, PROTACs are democratizing access to previously privileged targets and opening new avenues for treating rare diseases, cancer subtypes, and complex genetic disorders. The technology's unique ability to achieve catalytic, sub‐stoichiometric effects while potentially reducing off‐target toxicity positions it as a cornerstone of next‐generation precision medicine.

As we stand at the threshold of the first PROTAC drug approvals, the field faces the exciting challenge of translating this revolutionary technology into broad clinical benefit. The successful resolution of current technical limitations, combined with continued innovation in molecular design, delivery strategies, and clinical application, will determine whether PROTACs fulfill their transformative potential. The convergence of advancing scientific understanding, technological sophistication, and clinical validation suggests that TPD will become an indispensable tool in the modern therapeutic arsenal, offering hope for patients with currently intractable diseases and fundamentally changing how we approach drug discovery and development in the decades to come. The journey from the first PROTAC entering clinical trials in 2019 to potential approval in 2025 represents not merely a technological achievement, but the dawn of a new era in medicine where the impossible becomes possible through the elegant manipulation of cellular protein homeostasis.

## Author Contributions

M.L., Z.H.T., and J.Y. designed this study. G.F., S.L.C, Q.P.Z, N.Y. and Z.Y.S. collected related articles. G.F., S.L.C., Q.P.Z., N.Y., Z.Y.S., Z.J.L, and W.M.G. wrote the manuscript and completed the figures. M.L., Z.H.T., J.Y., and G.F. revised manuscripts and completed tables. G.F., Z.H.T. and J.Y. provided funding support. All authors reviewed and approved the final manuscript.

## Ethics Statement

The authors have nothing to report.

## Conflicts of Interest

The authors disclose no conflicts of interest.

## Data Availability

No data were used for the research described in the article.
