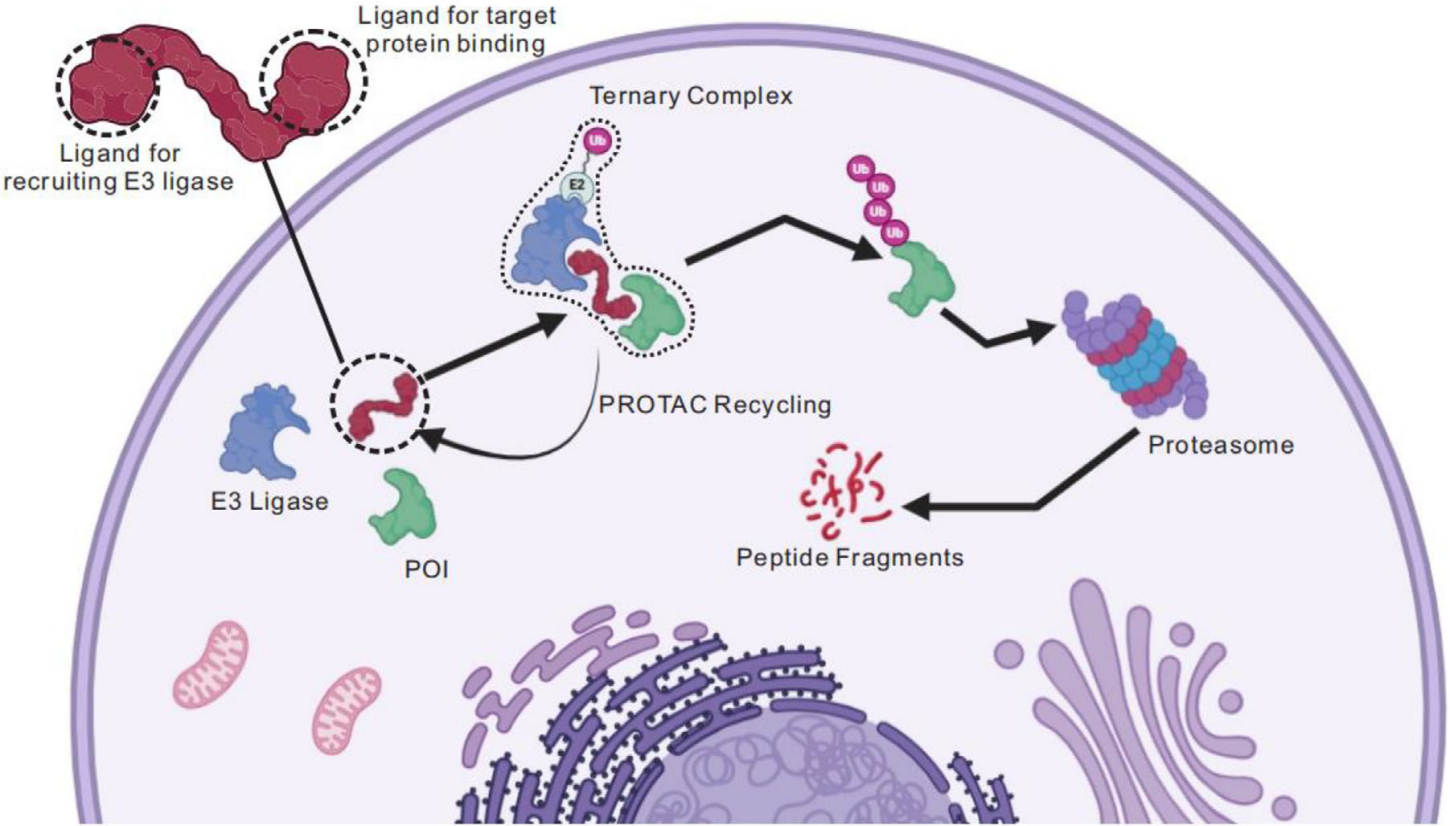# Correction to “Proteolysis‐targeting chimera (PROTAC): current applications and future directions”

**DOI:** 10.1002/mco2.70491

**Published:** 2025-11-05

G. Fan, S. Chen, Q. Zhang, et al., “Proteolysis‐Targeting Chimera (PROTAC): Current Applications and Future Directions,” MedComm 6, no. 10 (2025): e70401.

In Figure 1 of our published article, there is a minor typographical error in the labeling. The text currently reads “Ligase for recruiting E3 ligase” and “Ligase for target protein binding,” which should instead read “Ligand for recruiting E3 ligase” and “Ligand for target protein binding.” This correction does not affect the interpretation of the figure.

We apologize for this error.


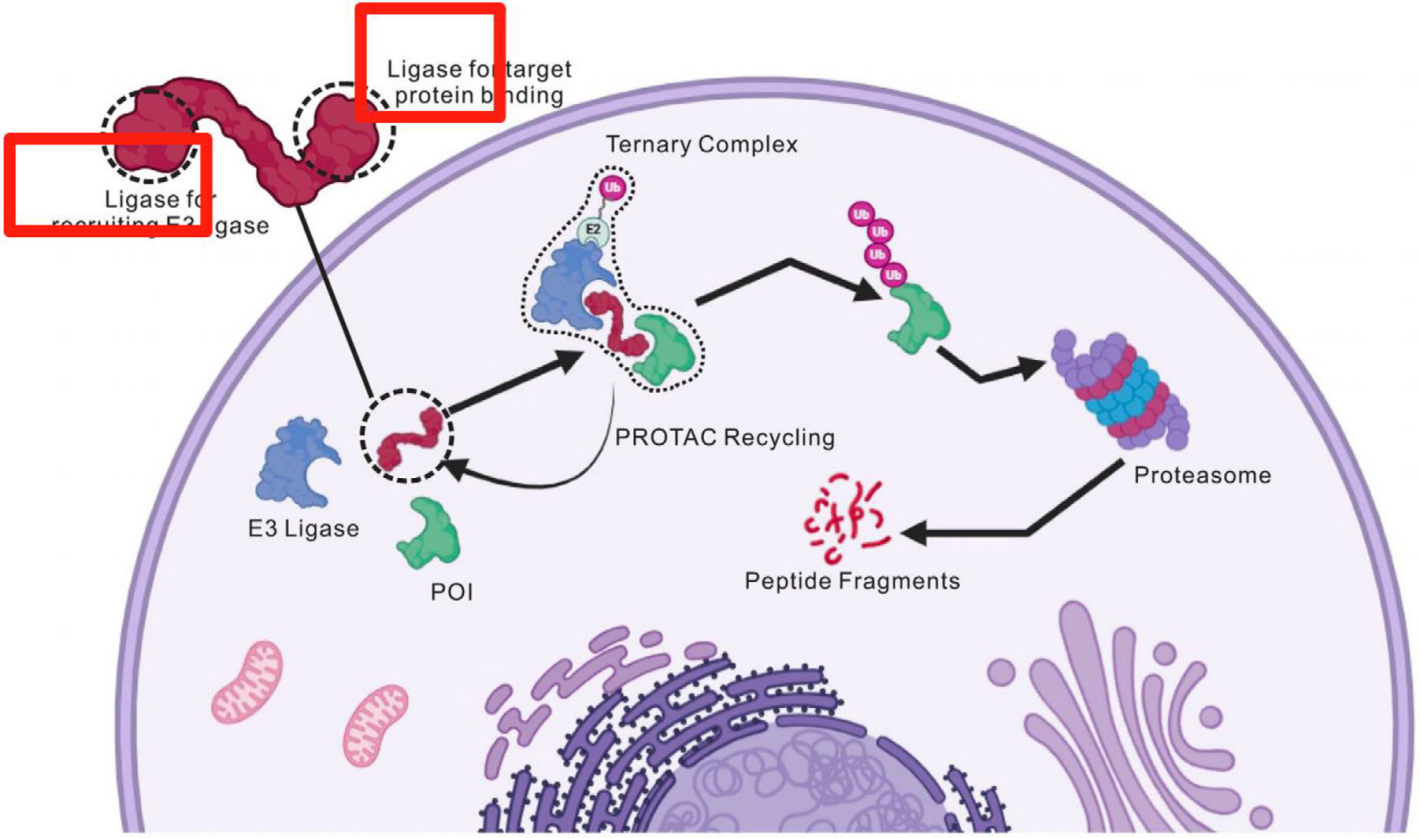


Modified as follow